# Complete Mapping of Thermodynamic Stability of Ternary Oxide SrTiO_3_ (001) Surface at Finite Temperatures

**DOI:** 10.1002/advs.202405450

**Published:** 2024-09-05

**Authors:** Md Mokhlesur Rahman, Sehoon Oh, Puspa Raj Adhikari, Jaichan Lee

**Affiliations:** ^1^ School of Advanced Materials Science & Engineering Sungkyunkwan University Suwon‐si Gyeonggi‐do 16419 South Korea

**Keywords:** electronic structure calculation, finite‐temperature‐DFT, perovskite oxides, surface reconstruction, thermodynamic stability

## Abstract

The oxide surface structure plays a vital role in controlling and utilizing the emergent phenomena occurring at the interface of nanoarchitecture. A complete understanding of ternary oxide surfaces remains challenging due to complex surface reconstructions in various chemical and physical environments. Here a thermodynamic framework is developed to treat the stability of ternary oxide surfaces with finite temperature and chemical environments. Strontium titanate, as a representative ternary oxide, is used to establish the complete energy landscape of SrTiO_3_ (001) surface. The complete mapping yields a comprehensive understanding of various stable SrTiO_3_ surfaces with finite temperature and chemical potential or vapor pressure of the constituents, i.e., Sr (or Ti) metal and oxygen. This treatment also reveals a stable surface unknown yet with SrTi_2_O_3_ stoichiometry, which unveils the missing link between numerous previous experimental observations and the current understanding of SrTiO_3_ surface. Interestingly, the new surface shows an anisotropic surface‐localized metallic state originating from the characteristic surface structure. The findings would provide a viable way to understand ternary oxide surfaces and further utilize SrTiO_3_ surfaces for oxide nanoarchitectures.

## Graphical abstract

Exploring the complex behavior of ternary oxide surfaces, this study introduces a thermodynamic framework to understand their stability under various temperatures and chemical conditions. By using strontium titanate as a model, we map the energy landscape of SrTiO_3_ (001) surfaces, uncovering a previously unknown stable surface. This surface exhibits unique stoichiometry and anisotropic metallic properties, offering new insights for the development of oxide nanoarchitectures.

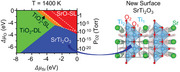

## 1 Introduction

Ternary oxide surfaces are widely studied experimentally and theoretically for their versatility in growing and assembling other nanomaterials into nanoarchitectures.^[^ ^1^, ^2^, ^3^, ^4^, ^5^ ^]^ Density functional theory (DFT) is a useful theoretical tool for investigating the thermodynamics of ternary oxide surfaces^[^ ^6^, ^7^, ^8^, ^9^ ^]^; however, it has limitations since it is usually performed at zero temperature without considering thermal contributions. Although the temperature and pressure dependence of the gas phase has been obtained using available empirical thermodynamic data^[^ ^10^ ^]^ or statistical mechanics,^[^ ^11^ ^]^ condensed state calculations are typically done at zero temperature or with approximated vibrational energy contributions of surface adsorbates.^[^ ^12^ ^]^ As a result, the outcomes of those computations cannot be applied to high‐temperature processing conditions such as growth and annealing in various chemical environments. The entropic contribution is reported to play a crucial role in binary oxide surfaces.^[^ ^13^ ^]^ The entropic contribution will also be important for ternary oxides, while its effect on the ternary oxide surface is yet to be understood properly. A framework is developed here to include the entropic contribution in the first‐principles calculations for treating the thermodynamic stability of ternary oxide surfaces at finite temperatures and in various chemical environments.

We study perovskite SrTiO_3_ (STO), an archetypal material used to produce 2D electron gases(2DEGs)^[^ ^2^, ^14^, ^15^, ^16^ ^]^ at oxide interfaces, which exhibit remarkable properties, including metal‐insulator transition,^[^ ^17^, ^18^ ^]^ superconductivity,^[^ ^19^, ^20^ ^]^ and large negative magnetoresistance.^[^ ^21^ ^]^ Given the extensive functionality of heterojunctions based on STO, oxide electronics could be a promising alternative to silicon‐based electronics. Thus, it is of fundamental importance to understand the STO surface to utilize its diverse functionality.

As‐grown STO (001) surface is composed of SrO‐terminated (SrO‐SL) and TiO_2_‐terminated (TiO_2_‐SL) surfaces.^[^ ^22^, ^23^, ^24^ ^]^ Annealing TiO_2_‐SL surfaces results in various surface reconstructions depending on the surrounding environment.^[^ ^8^, ^25^, ^26^, ^27^, ^28^, ^29^, ^30^, ^31^, ^32^, ^33^, ^34^, ^35^, ^36^, ^37^, ^38^, ^39^, ^40^, ^41^, ^42^, ^43^, ^44^ ^]^ Some reconstructed surfaces are TiO_2_‐rich, with no Sr ions present on the surface or sub‐surface (TiO_2_‐DL),^[^ ^25^, ^36^, ^38^ ^]^ while other studies have reported strong evidence of Sr ions on the surface.^[^ ^39^, ^40^, ^41^, ^42^, ^45^ ^]^ The variation in surface stoichiometry with the periodicity of the reconstructed surface is also reported.^[^ ^46^ ^]^ For the reconstructed surfaces with Sr ions, several candidate surface structures have been suggested, such as SrO oxide segregation,^[^ ^39^, ^41^ ^]^ SrO rock‐salt cluster formation,^[^ ^40^ ^]^ and micro‐faceting of TiO_2_‐SL with Sr adatom.^[^ ^42^ ^]^ Though these suggested surfaces have a common characteristic of ≈1 eV peak‐shift of Sr 3*d* spectra^[^ ^39^, ^40^, ^41^ ^]^ at high temperatures, the surface structure remains highly argued. Our comprehensive analysis of the STO (001) surface based on first‐principles calculations has shed new light on this argument by revealing a novel surface structure with a SrTi_2_O_3_ surface stoichiometry. Utilizing the proposed methodology, a complete energy landscape of STO (001) surface, i.e., temperature‐dependent thermodynamic phase diagram (TPD), has been obtained, which provides the appropriate chemical environment for the known and new surfaces at various temperatures. Through a careful analysis of the obtained TPD and the simulation results of the Sr peak‐shift, we conclude that the new surface is the unveiled reconstructed surface containing Sr atoms.

## 2 Results and Discussion

### 2.1 Exploring STO (001) Surfaces

**Figure 1:**
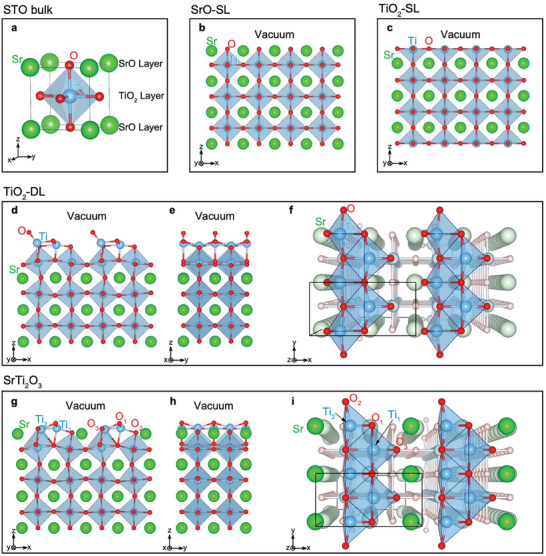
Atomic structures of STO (001) surfaces. The atomic structures of bulk STO a), SrO‐SL b) TiO_2_‐SL c), TiO_2_‐DL ‐d‐f), and SrTi_2_O_3_ g–i) surfaces are presented. Side view (b‐e,g, and h) and top view (f and i) are shown. The z‐direction is normal to the surface. The green, cyan, and red spheres represent the Sr, Ti, and O atoms, respectively, and the Ti‐centered octahedrons are shaded in cyan. In (f) and (i), the atoms not on the surface are blurred in order to focus on the surface structure, and black rectangles represent the (2 × 1) surface unit cells.

Bulk STO consists of alternating charge‐neutral SrO and TiO_2_ layers along three principal axes, and two different surface terminations, SrO‐SL and TiO_2_‐SL, are expected. Besides, reconstructed surfaces followed by thermal treatment are divided mainly into two groups. One is TiO_2_‐DL,^[^ ^25^, ^36^, ^38^ ^]^ where two adjacent TiO_2_ layers assemble as the surface and the sub‐surface. The other is a surface structure containing Sr ions,^[^ ^39^, ^40^, ^41^, ^42^ ^]^ whose surface geometry has not been elucidated yet. Considering these reports, we investigated the atomic structures of STO (001) surfaces using first‐principles calculations based on DFT. Based on the optimized atomic structure of STO bulk (**Figure** **1**a; Figure S1, Supporting Information), we constructed the surface structures of SrO‐SL and TiO_2_‐SL with vacuum, and the atomic positions of the constructed structures were relaxed by minimizing the total energy. The obtained atomic structures (Figure 1b,c) are consistent with previous reports (see Tables S1 and S2, Supporting Information).^[^ ^47^, ^48^ ^]^ For TiO_2_‐DL, a candidate surface structure was constructed based on the surface geometry of a previous report^[^ ^25^ ^]^ and optimized by minimizing the total energy. The optimized structure (Figure 1d–f) is consistent with previous reports (Table S3, Supporting Information).^[^ ^25^, ^40^ ^]^

We investigated all possible structures beyond the three known surfaces on a (2 × 1) surface unit cell, which is a basic building block for generating many surface reconstructions (Note S3, Supporting Information), and screened structures that do not follow any of the following conditions: i) The octahedron interstitial site should not be occupied by Sr or O atom. ii) A‐site should not be occupied by Ti atom. iii) O‐site should not be occupied by Sr or Ti atom. iv) Ti atoms should not be sandwiched by adjacent Sr atoms. v) The number of O atoms should not be less than that of Ti atoms. vi) The surface should contain at least two species of Sr, Ti, and O. vii) The B‐site should not be occupied by an O atom. As a result, 26 candidate surface structures with ten different stoichiometries are found to satisfy all the conditions, and their stabilities are estimated by DFT calculation. Among the 26 candidate structures, it is found that only one structure with SrTi_2_O_3_ surface stoichiometry on top of the TiO_2_ sub‐surface is thermodynamically stable (Notes S4 and S5, Figures S3–S5 and Tables S4–S6, Supporting Information). Figure 1g–i depicts the surface geometry of the obtained “SrTi_2_O_3_” surface. Sr atom and one O atom (O_1_) occupy the A‐sites, while other two O atoms (O_2_ and O_3_) are located at the O sites on top of the sub‐surface Ti atoms. Ti atoms with 4‐coordination (Ti_1_) and 5‐coordination (Ti_2_) are located at the octahedral interstitial sites on top of the sub‐surface O atoms. Note that the surface Ti atoms share their octahedral edges with each other and with the sub‐surface Ti atoms, which can be interpreted as the edge‐sharing mechanism of surface stabilization proposed in a previous study for TiO_2_‐DL.^[^ ^25^ ^]^

### 2.2 Thermodynamic Stability of Perovskite Oxide Surfaces

To include the entropic contribution in the first‐principles thermodynamic formalism,^[^ ^6^, ^7^, ^8^ ^]^ we define a surface formation energy per unit area *Ω^i^* (𝛥𝜇_Sr_,𝛥𝜇_O_;*T*) for surface *i (i* = TiO_2_‐SL, TiO_2_‐DL, SrO‐SL, and SrTi_2_O_3_), as a function of the chemical potentials of Sr (𝛥𝜇_Sr_) and O (𝛥𝜇_O_), and temperature *T*, by calculating the DFT total energy and the phonon correction (Note S6 and Figures S6–S8, Supporting Information). To start with, we define the surface formation energy, *E_f_*, as

(1) $$E_{f}\left(\mu_{x};T\right)\;=\;1/2\;\left[G_{\mathrm{slab}}\left(T\right)-\sum_{X}n_{X}\mu_{x}\left(T\right)\right]$$

where *G*_slab_ is the Gibbs free energy of the slab containing two identical surfaces on both sides, *n*_X_ is the number of atoms in the slab, 𝜇_x_ is the chemical potential of species X (X = Sr, Ti, and O). Here, *G*_slab_ is defined as,

(2) $$G_{\mathrm{slab}}\left(T\right)={E^{\mathrm{DFT}}}_{\mathrm{slab}}+{E^{\mathrm{vib}}}_{\mathrm{slab}}\left(T\right)-T{S^{vib}}_{slab}\left(T\right)$$

where *E* ^DFT^_slab_, *E* ^vib^_slab_, and *S* ^vib^_slab_(*T*) are the DFT total energy, the vibrational energy, and the vibrational entropy of the slab, respectively. The number of atoms on STO (001) surfaces nearly equals the number of available positions. As a result, the contribution of configurational entropy has been disregarded. We use the equilibrium condition, *g*_STO_(*T*) = 𝜇_Sr_(*T*)+𝜇_Ti_(*T*)+3𝜇_O_(*T*), where *g*_STO_ is the Gibbs free energy of the bulk STO per unit cell, which is defined as,

(3) $$g_{\mathrm{STO}}=\left[{E^{\mathrm{DFT}}}_{\mathrm{STO}}+{E^{\mathrm{vib}}}_{\mathrm{STO}}\left(T\right)-T{S^{\mathrm{vib}}}_{\mathrm{STO}}\left(T\right)\right]/N_{\mathrm{cell}}$$

where *E* ^DFT^_STO_, *E* ^vib^_STO_, *S* ^vib^_STO_(*T*), and *N*_cell_ are the DFT total energy, the vibrational energy, the vibrational entropy of STO bulk, and the number of unit cells in the calculation, respectively. With the equilibrium condition, the Equation. (1) becomes,

(4) $$\begin{array}{ccc} E_{f}\left(\mu_{\mathrm{Sr}},\mu_{O};T\right) & = & 1/2\;\left[G_{\mathrm{slab}}\left(T\right)-n_{\mathrm{Ti}}g_{\mathrm{STO}}\left(T\right)\right.\\ & & \left.-\;\left(n_{\mathrm{Sr}}-n_{\mathrm{Ti}}\right)\mu_{\mathrm{Sr}}\left(T\right)-\left(n_{O}-3n_{Ti}\right)\mu_{O}\left(T\right)\right] \end{array}$$

By introducing new variables, 𝛥𝜇_X_ = 𝜇_X_–𝜇^0^_X_, we get the final expression for surface formation energy (*Ω^i^*),

(5) $$\begin{array}{ccc} & & \Omega^{i}\left(\Delta \mu_{\mathrm{Sr}},\Delta \mu_{O};T\right)=E_{f}\left(\Delta \mu_{\mathrm{Sr}},\Delta \mu_{O};T\right)/A=1/\left(2A\right)\\ & & \;\left[G_{\mathrm{slab}}\left(T\right)-n_{\mathrm{Ti}}g_{\mathrm{STO}}\left(T\right)-\left(n_{\mathrm{Sr}}-n_{\mathrm{Ti}}\right)\;\left(\Delta \mu_{\mathrm{Sr}}\left(T\right)+{\mu^{0}}_{\mathrm{Sr}}\left(T\right)\right)\right.\\ & & \;\left.-\left(n_{O}-3n_{Ti}\right)\;\left(\Delta \mu_{O}\left(T\right)+{\mu^{0}}_{O}\left(T\right)\right)\right] \end{array}$$

where 𝜇^0^_Sr_ and 𝜇^0^_O_ are the chemical potentials of Sr bulk and O_2_ molecule, respectively, and *A* is the surface area. Here, 𝜇^0^_X_(*T*) is defined as, 𝜇^0^_X_(*T*) = (*E* ^DFT^_X_+*E* ^vib^_X_(*T*)–*TS* ^vib^_X_(*T*))/*N*_X_, where *N*_X_ is the number of X atoms in the unit cell. The vibrational energy *E* ^vib^(*T*), and the vibrational entropy, *S* ^vib^(*T*) for the solids and O_2_ molecule are obtained using the equations,

(6) $$E^{\mathrm{vib}}\left(T\right)={\Sigma_{q}}_{,s}\hbar {\omega_{q}}_{,s}\left({n_{q}}_{,s}\left(T\right)+1/2\right)$$

and

(7) $$\begin{array}{ccc} S^{\mathrm{vib}}\left(T\right) & = & k_{B}{\Sigma_{q}}_{,s}ln\left[2\sinh\left(\hbar {\omega_{q}}_{,s}/2k_{B}T\right)\right]\\ & & +\;1/\left(2T\right){\Sigma_{q}}_{,s}\hbar {\omega_{q}}_{,s}\coth\left(\hbar {\omega_{q}}_{,s}/2k_{B}T\right) \end{array}$$

respectively, where *k*_B_ is the Boltzmann constant, *ħ* is the reduced Plank constant, *ω_q_*_,_ *_s_* is the calculated frequency of the phonon with momentum *q* and band index *s*, and *n_q_*_,_ *_s_* (*T*) = [*exp*(*ħω_q_*_,_ *_s_*/*k*_B_ *T*)–1]^−1^ is the occupation function for the phonon. To obtain the thermodynamically allowed conditions for perovskite STO, we use five boundary conditions (BCs):

- 𝛥𝜇_Sr_(*T*) ⩽ 0; for non‐precipitation of Sr metal,
- 𝛥𝜇_O_(*T*) ⩽ 0; for non‐evaporation of O_2_ molecule,
- 𝛥𝜇_Sr_(*T*) + 3𝛥𝜇_O_(*T*) ⩾ 𝛥*g*_STO_(*T*); for non‐precipitation of Ti metal,
- 𝛥𝜇_Sr_(*T*) + 𝛥𝜇_O_(*T*) ⩾ 𝛥*g*_STO_(*T*) – 𝛥*g*_TiO2_(*T*); for non‐precipitation of SrO bulk, and
- 𝛥𝜇_Sr_(*T*) + 𝛥𝜇_O_(*T*) ⩽ 𝛥*g*_SrO_(*T*); for non‐precipitation of TiO_2_ bulk,

where, 𝛥*g*_STO_(*T*) = *g*_STO_(*T*) – 𝜇^0^_Sr_(*T*) – 𝜇^0^_Ti_(*T*) – 3𝜇^0^_O_(*T*), 𝛥*g*_SrO_(*T*) = *g*_SrO_(*T*) – 𝜇^0^_Sr_(*T*) – 𝜇^0^_O_(*T*), 𝛥*g*_TiO2_(*T*) = *g*_TiO2_(*T*) – 𝜇^0^_Ti_(*T*) – 2𝜇^0^_O_(*T*), *g*_SrO_(*T*) = [*E* ^DFT^_SrO_ + *E* ^vib^_SrO_(*T*) – *TS* ^vib^_SrO_(*T*)] / *N*_cell_, and *g*_TiO2_(*T*) = [*E* ^DFT^_TiO2_ + *E* ^vib^_TiO2_(*T*) – *TS* ^vib^_TiO2_(*T*)] / *N*_cell_.

**Figure 2:**
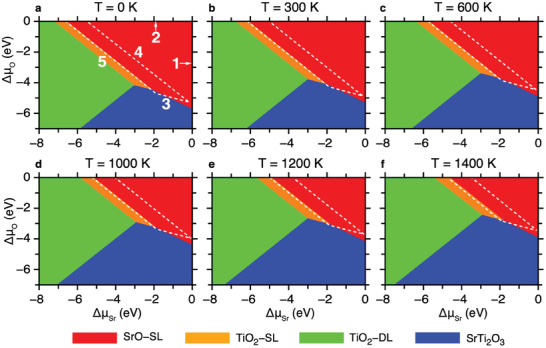
Temperature‐dependent TPD of STO (001) surface at various temperatures. TPD shows the most stable surface as a function of 𝛥𝜇Sr and 𝛥𝜇O at temperature 0 K a), 300 K b), 600 K c), 1000 K d), 1200 K e), and 1400 K f). The allowed region of the bulk perovskite STO is represented by white dashed lines, where lines 1–5 correspond to BCs (i)‐(v), respectively. BC (i): the non‐precipitation limit of Sr metal, BC (ii): the non‐evaporation limit of O_2_ molecule, BC (iii): the non‐precipitation limit of Ti metal, BC (iv): the non‐precipitation limit of SrO bulk, and BC (v): the non‐precipitation limit of TiO_2_ bulk.

We obtain the temperature‐dependent TPD of STO (001) surface by comparing *Ω^i^* s and using the BCs as shown in **Figure** **2**. Figure 2a–f shows the obtained TPD at 0, 300, 600, 1000, 1200, and 1400 K, respectively, presenting the most stable surface structure as a function of 𝛥𝜇_Sr_ and 𝛥𝜇_O_. The thermodynamically allowed region of the bulk STO is enclosed by lines 1–5 corresponding to the BC (i)‐(v), respectively. Only three surfaces, SrO‐SL, TiO_2_‐SL, and SrTi_2_O_3_, appear in the allowed region, whereas TiO_2_‐DL is outside the region, which we will discuss below. SrO‐SL occupies the largest area of the allowed region, followed by TiO_2_‐SL and SrTi_2_O_3_. Interestingly, the obtained TPD is strongly dependent on temperature. With increasing temperature, the allowed region and the boundaries between the surfaces shift to the O‐rich region; thus, SrTi_2_O_3_ stabilizes at higher 𝛥𝜇_O_. The TPD is replotted as a function of *P*_O2_ and 𝛥𝜇_Sr_ using 𝛥𝜇_O_ = *k*_B_ *T ln*(*P*_O2_/*P* ^0^),^[^ ^6^, ^7^ ^]^ where *P* ^0^ is the standard pressure (1 atm). At 300 and 600 K, SrTi_2_O_3_ stabilizes at *P*_O2_ = ≈10^−70^ and ≈10^−29^ Torr, respectively. As temperature increases, SrTi_2_O_3_ stabilizes in experimentally accessible conditions such as annealing temperatures widely reported and oxygen environments: *P*_O2_ = ≈10^−14^ Torr at 1000 K, ≈10^−10^ Torr at 1200 K, and ≈10^−8^ Torr at 1400 K (Note S7 and Figure S9, Supporting Information). We examined the contribution of phonon energy and entropy separately on the stability of these four surfaces and found that phonon energy favors the stability of SrO‐SL and TiO_2_‐SL surfaces, whereas entropic contribution favors the thermodynamic stability of SrTi_2_O_3_ surfaces (See Figures S11 and S12 and Note S9, Supporting Information). At low temperatures, the phonon energy is dominant over the entropic contribution, while at high temperatures, the entropic contribution becomes dominant over phonon energy (Figure S13, Supporting Information). Experimentally found TiO_2_‐DL has water absorption on it,^[^ ^49^ ^]^ which has a lower surface formation energy compared to dry TiO_2_‐DL.^[^ ^50^ ^]^ In this study, water absorption is not taken into account, which we believe is the reason why TiO_2_‐DL is outside the allowed region. Considering the similarity between the surface structures of TiO_2_‐DL and SrTi_2_O_3_ (Figure 1d–i), water molecules can be adsorbed on SrTi_2_O_3,_ lowering the surface formation energy of SrTi_2_O_3_ further. Calculating the stability of the water‐absorbed TiO_2_‐DL and SrTi_2_O_3_ surfaces would be helpful, but it is beyond the scope of this work.

### 2.3 The XPS Simulation

**Figure 3:**
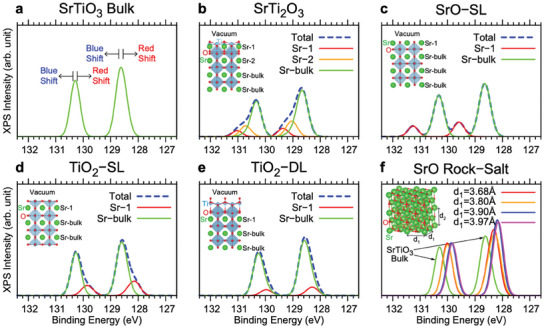
Simulated XPS spectra of Sr 3*d* core‐level. The XPS spectra of Sr core‐level are simulated in the initial state approximation by calculating the binding energies of Sr 3*d*_5/2_ and 3*d*_3/2_ core‐levels using first‐principles DFT calculations. The obtained XPS spectra of the core‐levels for STO bulk a), SrTi_2_O_3_ b), SrO‐SL c), TiO_2_‐SL d), TiO_2_‐DL e) surfaces, and SrO rock‐salt bulk f) are shown. In (b‐e), the simulated XPS spectra are represented by blue dashed lines, and the spectra contributed from the bulk parts and those from the topmost Sr atoms (denoted by Sr‐1 in the insets), are separately plotted and represented by green and red lines, respectively. In (b), the simulated spectra from the 2^nd^ topmost Sr atoms (denoted by Sr‐2 in the inset), which is off both the bulk and surface peak positions, are separately plotted and represented by yellow lines. In (f), the simulated Sr core‐level XPS spectra of the elongated SrO rock‐salt are shown. The atomic structures of elongated rock‐salt SrO are obtained by minimizing the total energy varying the out‐of‐plane lattice constant, *d_2_*, with the in‐plane lattice constant, *d_1_*, fixed, where *d_1_* = 3.68 Å and *d_1_* = 3.97 Å corresponds to the lattice constants of the cubic rock‐salt and STO bulk, respectively. The spectra of the STO bulk are presented by green lines for comparison.

Following the discussion on the stability of the surfaces, we bring up the long‐debated question of the geometry of the reconstructed surface upon annealing. We would start the discussion by summarizing experimental observations. When the surface is annealed at high *P*_O2_, it becomes a TiO_2_‐rich surface without containing Sr ions, which is identified to be TiO_2_‐DL.^[^ ^25^, ^36^, ^38^ ^]^ In contrast, when annealed at low *P*_O2_, Sr ions are observed on the surface.^[^ ^39^, ^40^, ^41^, ^42^, ^45^ ^]^ During the annealing at low *P*_O2_, the Sr coverage on the surface increases with ≈1 eV peak‐shift of the Sr core‐level spectra, which was previously interpreted as an effect of the elongated Sr‐oxide formed on the TiO_2_‐rich surface.^[^ ^40^ ^]^ Thus, we investigate the peak‐shifts of the Sr 3*d* core states of the STO surfaces, and those of the SrO rock‐salt structures using DFT calculations. We simulate the XPS spectra of Sr 3*d*_5/2_ and 3*d*_3/2_ core‐levels as shown in **Figure** **3**. The calculated binding energies for SrTi_2_O_3_ (Figure 3b) and SrO‐SL (Figure 3c) increase (blue shift) compared to that of bulk (Figure 3a), whereas those for TiO_2_‐SL (Figure 3d) and TiO_2_‐DL (Figure 3e) decrease (redshift). The calculated blue shifts for SrTi_2_O_3_ and SrO‐SL are 0.8 and 0.9 eV, respectively, and both are in good agreement with the experimentally observed value of ≈1 eV.^[^ ^39^, ^40^, ^41^ ^]^ We attribute this large peak‐shift to the asymmetric charge distribution near the surface Sr ion mainly due to the lack of other ions above the Sr ion, i.e., the coordination number changes.

Figure 3f shows the simulated spectra of the Sr core‐states in SrO rock‐salt bulk with elongation. The peak positions of the cubic (*d*_1_ = 3.68 Å) and elongated (*d*_1_ = 3.97 Å) SrO show ≈0.2 and ≈0.4 eV red shifts compared to those of STO bulk, respectively. The direction of the peak‐shift is not only opposite, but the magnitude is also too small to explain the blue shift observed in previous experimental studies.^[^ ^39^, ^40^, ^41^ ^]^ This suggests that the formation of SrO rock‐salt cannot be the cause of the blue shift noted in these earlier experiments.^[^ ^39^, ^40^, ^41^ ^]^ We emphasize that significant peak‐shifts of the spectra can be explained by a significant change of the charge distribution, such as a change in the coordination number, NOT by a small change due to the elongation. Moreover, it is reported that the Sr coverage increases to 20–25% during the annealing while the Ti coverage does not decrease,^[^ ^40^, ^45^ ^]^ eliminating the possibility of the peak‐shift occurring on the SrO‐SL. If the surface had converted to SrO‐SL, we would have observed a reduction in Ti coverage, which did not happen. Thus, we conclude that the observed large blue shift should originate from SrTi_2_O_3_.

### 2.4 Process of Surface Reconstructions

Based on the experimental observations and the analysis of the peak‐shift simulation, we propose a process of how the surface is reconstructed during the annealing depending on *P*_O2._ For high *P*_O2_, all surface area becomes TiO_2_‐DL without Sr atom on the surface. For low *P*_O2_, some Sr atoms remain on the surface, and the surface area containing the Sr atoms becomes SrTi_2_O_3_, while the surface area not containing Sr atoms becomes TiO_2_‐DL, forming a mixture of SrTi_2_O_3_ and TiO_2_‐DL areas. Further annealing at low *P*_O2_ increases the SrTi_2_O_3_ area by replacing O atoms on the TiO_2_‐DL area with Sr atoms, and thus increasing the Sr coverage on the surface (Note S10 and Figure S14, Supporting Information). We examine the validity of our proposed process by comparing the stabilities of TiO_2_‐DL and SrTi_2_O_3_ (Figure S15, Supporting Information). With high *P*_O2_, TiO_2_‐DL is more stable than SrTi_2_O_3_ for all *T* investigated. With low *P*_O2_, TiO_2_‐DL is more stable than SrTi_2_O_3_ at low *T*, whereas SrTi_2_O_3_ becomes more stable than TiO_2_‐DL at high *T*, consistent with the experimental reports.^[^ ^40^, ^45^ ^]^ The transition temperature between the two surfaces decreases as *P*_O2_ decreases, consistent with experiments.^[^ ^40^, ^45^ ^]^

### 2.5 The Electronic and Magnetic Structures of the SrTi_2_O_3_ Surface

**Figure 4:**
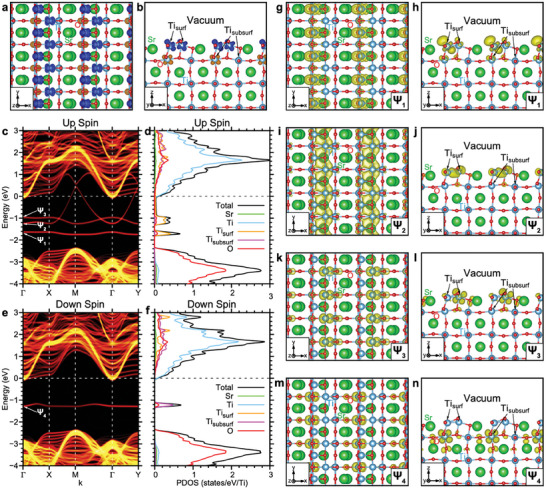
Electronic Structures of SrTi_2_O_3_ surface. The electronic structures of SrTi_2_O_3_ surface are shown. The spin density of the ferrimagnetic configurations with the atomic structure a,b). The isosurfaces of the positive (for majority spin) and negative (for minority spin) values of the spin density, which is defined as the difference between the charge densities of the majority (up) and minority (down) spin components, are shaded in blue and orange, respectively. The electronic band structures and the projected density of states of the surface c–f). The electronic structures for the majority (c,d) and minority (e,f) spins of the ferrimagnetic configuration are presented. In (c–f), the Fermi energy is set to be zero and marked with horizontal dashed lines. In c and e, the bands are unfolded with respect to the first Brillouin zone of the (1×1) surface unit cell, where the denoted high‐symmetry points are Γ = 0, X = π/*a* $\hat{x}$, Y = π/*a* $\hat{y}$, and M = π/*a* ($\hat{x}$+$\hat{y}$) with the lattice constant *a* = 3.972 Å. In d and f, the total density of states is plotted by black lines, and the density of states projected onto Sr, Ti, and O atoms are plotted by green, cyan, and red lines, respectively. The densities of states projected onto Ti atoms on the surface (denoted as Ti_surf_) and those on the sub‐surface (denoted as Ti_subsurf_) are separately plotted by yellow and magenta lines, respectively. The wavefunctions of the states between the valence and conduction bands (denoted as ψ_1_ to ψ_4_) of the ferrimagnetic configuration g‐n). The wavefunctions are presented in real space with the atomic structure. The top view (g,i,k, and m) and side view (h,j,l, and n) are shown and the isosurfaces for the squared wavefunctions of the states are shaded in yellow.

We also investigate the electronic and magnetic properties of SrTi_2_O_3_. We calculate the electronic structures with various magnetic configurations, but the total energy differences between different magnetic structures are up to 10 meV/unit surface area, implying no stable magnetic structure at high temperatures. The ground state is a ferrimagnetic configuration, where the magnetic moments of all surface Ti atoms (Ti_surf_) have the same direction while only half of the sub‐surface Ti atoms (Ti_subsurf_) carry finite magnetic moments anti‐parallel to those of the Ti_surf_ (**Figure** **4**a,b). Figure 4c–f shows the obtained electronic structure of the ferrimagnetic ground state. We find surface states between the energies of the valence band maximum (*E*_VBM_) and the conduction band minimum (*E*_CBM_). For the majority spin, three surface states are found between *E*_VBM_ and *E*_CBM_ (Figure 4c,d). Two flat bands, Ψ_1_ and Ψ_3_, are localized states (Figure 4g,h,k,l), while the other band, Ψ_2_, mainly consisting of the *d*‐orbitals of Ti_surf_s (Figure 4i,j), is dispersive crossing the Fermi level through the electron hopping between Ti_surf_s along the *y*‐direction, while flat along the *x*‐direction (Figure 4c). For minority spin, only one localized flat band, Ψ_4_, is found between *E*_VBM_ and *E*_CBM_ (Figure 4e,f,m,n). Note that the existence of the dispersive band and the subsequent metallic behavior of the SrTi_2_O_3_ surface do not depend on the magnetic configuration or calculation parameters such as Coulomb repulsion U (Note S11 and Figures S16–S18, Supporting Information). The dispersive band, connecting the bonding and antibonding states between Ti_surf_s, originates from the characteristic surface structure and the subsequent electron hopping between the atoms regardless of the calculation parameters. Therefore, SrTi_2_O_3_ surface is featured by the surface localized anisotropic dispersive band, Ψ_2_, making the surface metallic with strong anisotropy while the bulk part remains insulating.

## 3 Conclusion

A thermodynamic framework has been established to study ternary oxide surfaces. A comprehensive mapping of surface structure and stoichiometry with chemical environment and temperature is established for the STO (001) surface. The energy landscape of STO (001) surface reveals a new thermodynamically stable surface with SrTi_2_O_3_ surface stoichiometry on top of the TiO_2_ sub‐surface layer. The obtained TPD and the simulations of the Sr peak‐shift indicate that the new surface is the unveiled reconstructed surface containing Sr atoms after the thermal treatment. We provide a solution for how the surface is reconstructed during the etching and annealing, including the complex behavior of Sr accommodation on the surface, which has remained a matter of dispute for decades. The electronic structure of the new surface shows an interesting metallic surface state due to the electron hopping between the surface Ti atoms. Our findings would provide a comprehensive picture of STO surface, which can be utilized in the science and technology of oxide electronics toward finding novel functionality or engineering oxide architectures of various forms.

## 4 Experimental Section

### The First‐Principles DFT Calculation

The first‐principles DFT calculation was performed using the Vienna *ab‐initio* simulation package (VASP).^[^ ^51^, ^52^, ^53^ ^]^ The projected augmented wave (PAW)^[^ ^54^ ^]^ and generalized gradient approximation with Perdew–Burke–Ernzerhof scheme^[^ ^55^ ^]^ was used to treat the electron‐ion potential and exchange and correlation effects, respectively. The plane wave cut‐off energy of 850 eV was used. The ionic convergence criterion of |1 × 10^−3^| eV/ Å for Hellman‐Feynman forces was used with tetrahedron method with Blöchl corrections for the relaxation of lattice parameter and internal coordination. Γ‐centered 8 × 8 × 8 *k*‐point mesh was used for the self‐consistent calculations of STO bulk, Γ‐centered 8 × 8 × 1 mesh for the SrO‐SL and TiO_2_‐SL surfaces with (1 × 1) surface unit cell, and Γ‐centered 4 × 8 × 1 mesh for TiO_2_‐DL and candidate surface structures with (2 × 1) surface unit cell including SrTi_2_O_3_. Γ‐centered 32 × 64 × 1 *k*‐point mesh was used to calculate the PDOS of SrTi_2_O_3_. To correct the Coulomb repulsion of Ti‐3*d*, Liechtenstein's method^[^ ^56^ ^]^ implemented with the PAW method was employed. The effective Hubbard *U* (*U*_eff_) = 4.34 eV (5–0.64 eV) for Ti‐3*d* orbitals^[^ ^57^, ^58^, ^59^, ^60^ ^]^ was adopted in this study. Spin‐polarized calculations have been performed for all the calculations. It is found that the optimized bulk STO with a lattice parameter of 3.972 Å, and this value has been used throughout the calculations. A symmetric slab structure of 9‐unit cell thick (18 layers) along STO (001) direction has been modeled to avoid any fictitious electric field between the slabs with ≈23 Å of vacuum.

### The First‐Principles Phonon Calculation

The first‐principles phonon calculations with the finite displacement method^[^ ^61^, ^62^ ^]^ have been performed for free energy calculations at finite temperatures as implemented at Phonopy.^[^ ^63^ ^]^ Dynamical matrices were obtained using the calculated force constants of the structures with displacement amplitude of 0.01 Å and diagonalized to obtain the phonon frequency, *ω_q_*_,_ *_s_*, where *q* and *s* were the momenta and the band indices, respectively. Non‐analytical term correction has been applied to the dynamical matrix to treat the long‐range interaction of macroscopic electric fields.^[^ ^64^, ^65^ ^]^ For Sr bulk, Ti bulk, SrO bulk, and TiO_2_ bulk, the face‐centered cubic structure with four atoms, the hexagonal structure with three atoms, the cubic structure with eight atoms, and the tetragonal structure with six atoms in the unit cell were used for the atomic structure optimization, respectively. The (3 × 3 × 3) bulk supercells and the (2 × 2) surface supercells were used for the phonon calculations. For the phonon calculations, (64 × 64) *q*‐point mesh was used for the surfaces, and (64 × 64 × 64), (42 × 42 × 42), (54 × 54 × 88), (48 × 48 × 48), and (54 × 54 × 84) *q*‐point meshes were used for STO bulk, Sr bulk, Ti bulk, SrO bulk, and TiO_2_ bulk, respectively.

### The XPS Simulation

The XPS spectra of Sr 3*d*_5/2_ and 3*d*_3/2_ core levels were simulated in the initial state approximation^[^ ^66^ ^]^ by calculating the binding energies of the 3*d* core states using SIESTA^[^ ^67^ ^]^ within the semi‐core approach with the spin‐orbit interaction^[^ ^68^, ^69^ ^]^ included.

## Conflict of Interest

The authors declare no conflict of interest.

## Author Contributions

M.M.R. and S.O. contributed equally to this work. J.L. conceived the idea of the research. M.M.R. and P.R.A. searched for the surface structures and carried out the geometric screening of the candidate structures of the surface. M.M.R., S.O., and P.R.A. performed DFT calculations for the stability including the phonon calculations. S.O. carried out the simulation on XPS spectra of Sr 3*d* core‐level. M.M.R. and S.O. computed the electronic structures. J.L. supervised the project. All authors contributed to the discussion of the results and writing of the manuscript.

## Supporting information

Supporting Information

## Data Availability

The data that support the findings of this study are available in the supplementary material of this article.
