# Supplementary material for: Complete Mapping of Thermodynamic Stability of Ternary Oxide SrTiO3 (001) Surface at Finite Temperatures
Source: Adv Sci (Weinh). 2024 Sep 5;11(41):2405450. doi: 10.1002/advs.202405450 (PMC11538646; doi:10.1002/advs.202405450)
Supplement: Supplementary file 1 — Supporting Information [file ADVS-11-2405450-s001.docx]

Supporting Information

Complete mapping of Thermodynamic Stability of Ternary Oxide SrTiO_3_ (001) Surface at Finite Temperatures

*Md Mokhlesur Rahman^†^, Sehoon Oh^†^, Puspa Raj Adhikari, and Jaichan Lee**

†These authors contributed equally

Md Mokhlesur Rahman, Sehoon Oh, Puspa Raj Adhikari, and Jaichan Lee

School of Advanced Materials Science & Engineering

Sungkyunkwan University

Suwon, Gyeonggi-do, 16419, Korea

*E-mail: [jclee@skku.edu](mailto:jclee@skku.edu).

Keywords: Perovskite Oxide, Surface reconstruction, Thermodynamic stability, Finite-temperature-DFT, XPS simulation, Electronic structure calculation.

**Note S1. Atomic and electronic structure of bulk SrTiO_3_ (STO)**

STO is a perovskite (ABO_3_) oxide, having a cubic structure at room temperature. We optimize the atomic structure of the cubic bulk STO and the optimized lattice parameter is 3.972 Å. We calculate the electronic structure of the optimized bulk STO (Figure S1), consistent with previous study^[1]^.


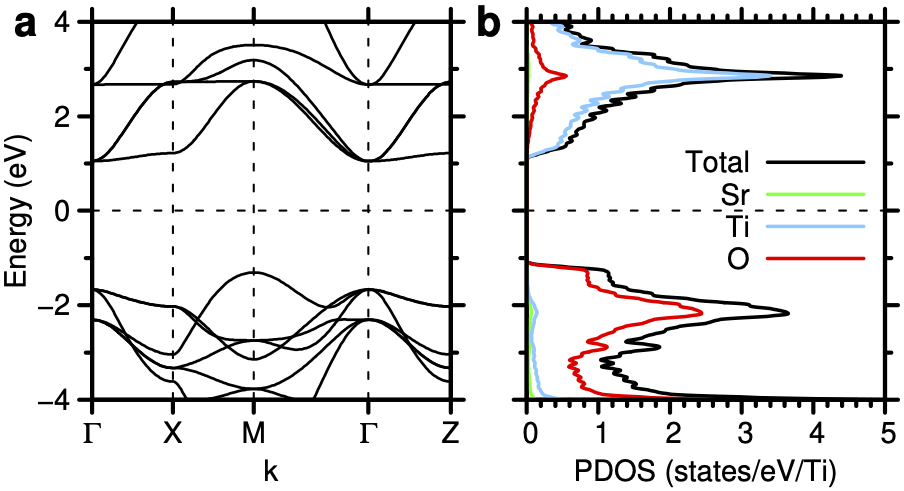


**Figure S1.** Electronic structure of bulk STO. The calculated electronic band structures (a) and the projected density of states (b) of the bulk STO are shown. In (a), the denoted high-symmetry points are Γ=0, X=π/*a* $\hat{x}$, Z=π/*a* $\hat{z}$, and M=π/*a* ($\hat{x}$+$\hat{y}$) with the optimized lattice constant *a* = 3.972 Å. In (b), the total density of states is plotted by black lines, and the density of states projected onto Sr, Ti, and O atoms are plotted by green, cyan, and red lines, respectively.

**Note S2. Relaxed atomic structures of SrO-SL, TiO_2_-SL, and TiO_2_-DL surfaces**

Relaxed atomic structures and comparison with references (if available) for SrO-SL (Table S1), TiO_2_-SL (Table S2), and TiO_2_-DL (Table S3) are presented below.

**Table S1.** Relaxed atomic positions of the SrO-SL surface structure. Calculated atomic displacements (relative to the ideal positions) for the SrO-SL surface are presented and compared with literatures. Units are in percent of the lattice constant *a*. Positive (negative) value of Δ*z* is for the displacements in the direction outward (inward) from the surface.

| Layers | Atoms | Atomic displacements [respect to the bulk lattice constant(*a*) in (%)] | | | |
| --- | --- | --- | --- | --- | --- |
|  |  | This work  GGA+U  *a*= 3.972 Å | Ref 1  LDA  *a*= 3.86 Å | Ref 2  LDA  *a*= 3.86 Å |  |
|  |  | Δ*z* | Δ*z* | Δ*z* |  |
| Layer-1 (Surface) | Sr 1 | –5.50 | –5.70 | –4.91 |  |
|  | O 1 | 0.13 | 0.10 | 0.92 |  |
| Layer-2  (Sub-surface) | Ti 1 | 1.20 | 1.20 | 1.20 |  |
|  | O 2 / O 3 | 0.19 | 0.00 | 0.48 |  |
| Layer-3 | Sr 2 | –1.51 | –1.12 | N/A |  |
|  | O 4 | –0.21 | –0.10 | N/A |  |
| Layer-4 | Ti 2 | 0.04 | N/A | N/A |  |
|  | O 5 / O 6 | –0.17 | N/A | N/A |  |
| Layer-5 | Sr 3 | –0.44 | N/A | N/A |  |
|  | O 7 | –0.18 | N/A | N/A |  |

*Ref 1: J. Padilla et al.,*^[2]^

*Ref 2: E. Heifetsat et al.,*^[3]^

**Table S2.** Relaxed atomic positions of the TiO_2_-SL surface structure. Calculated atomic displacements (relative to the ideal positions) for the TiO_2_-SL surface are presented and compared with literatures. Units are in percent of the lattice constant *a*. Positive (negative) value of Δ*z* is for the displacements in the direction outward (inward) from the surface.

| Layers | Atoms | Atomic displacements [respect to the bulk lattice constant(*a*) in (%)] | | |
| --- | --- | --- | --- | --- |
|  |  | This work  GGA+U  *a*= 3.972 Å | Ref 1  LDA  *a*= 3.86 Å | Ref 2  LDA  *a*= 3.86 Å |
|  |  | Δ*z* | Δ*z* | Δ*z* |
| Layer-1 (Surface) | Ti 1 | –2.82 | –3.40 | –2.12 |
|  | O 1 / O 2 | –0.77 | –1.60 | –1.11 |
| Layer-2  (Sub-surface) | Sr 1 | 3.25 | 2.50 | 2.21 |
|  | O 3 | –0.13 | –0.50 | 0.07 |
| Layer-3 | Ti 2 | –0.80 | –0.70 | N/A |
|  | O 4 / O 5 | –0.39 | –0.50 | N/A |
| Layer-4 | Sr 2 | –0.03 | N/A | N/A |
|  | O 6 | –0.20 | N/A | N/A |
| Layer-5 | Ti 3 | –0.28 | N/A | N/A |
|  | O 7 / O 8 | –0.20 | N/A | N/A |

Ref 1: J. Padilla *et al*.,^[2]^

Ref 2: E. Heifetsat *et al*.,^[3]^

**Table S3.** Relaxed atomic positions of the TiO_2_-DL (2×1) surface structure. Calculated atomic displacements (relative to the ideal positions) for the TiO_2_-DL surface are presented and compared with literatures. The *z*-position of the atoms on the 6^th^ layer from the surface ideal atomic position is assigned to 0. Positive (negative) value of Δ*z* is for the displacements in the direction outward (inward) from the surface. For Δ*x*, see Figure 1 in the main article for the convention of the in-plane axes.

| Layers | Atoms | Ideal position  *x*, *y*, *z*  [*a*] | Atomic displacements [respect to the lattice constant (*a*) in %] | | | | | | |
| --- | --- | --- | --- | --- | --- | --- | --- | --- | --- |
|  |  |  | This work  GGA+U  *a*= 3.972 Å | | Ref 3  GGA  *a*= 3.905 Å | | | Ref 4  GGA  *a*= 3.9045 Å | |
|  |  |  | Δ*x* | Δ*z* | Δ*x* | Δ*z* | Δ*x* | | Δ*z* |
| Layer-1  (surface) | Ti 1 | 1/2, 0, 5/2 | –6.75 | 9.78 | –0.22 | 9.10 | –5.28 | | 11.47 |
|  | Ti 2 | 1/2, 1/2, 5/2 | 3.67 | 3.42 | 4.10 | 3.80 | 4.10 | | 3.94 |
|  | O 1 | 0, 0, 5/2 | 17.98 | 42.92 | 14.04 | 38.07 | 21.37 | | 45.81 |
|  | O 2 | 1, 0, 5/2 | –6.16 | 15.81 | –5.84 | 16.63 | –5.25 | | 16.83 |
|  | O 3 | 1/2, 1/2, 5/2 | –1.70 | –2.57 | –0.92 | –1.88 | –1.22 | | –0.98 |
|  | O 4 | 3/2, 1/2, 5/2 | 0.08 | –0.71 | 0.58 | –0.35 | 0.29 | | 0.79 |
| Layer-2  (sub-surface) | Ti 3 | 1/2, 1/2, 2 | –3.22 | –2.35 | –2.92 | –0.74 | –3.59 | | –1.23 |
|  | Ti 4 | 3/2, 1/2, 2 | 2.31 | –1.02 | 2.80 | 1.79 | 2.93 | | 0.93 |
|  | O 5 | 0, 1/2, 2 | –0.19 | –9.33 | 0.02 | –7.12 | –0.16 | | –7.56 |
|  | O 6 | 1, 1/2, 2 | –0.66 | 5.62 | –0.14 | 7.55 | 0.11 | | 7.09 |
|  | O 7 | 1/2, 0, 2 | –4.53 | 3.40 | –4.58 | 5.79 | –4.08 | | 4.34 |
|  | O 8 | 3/2, 0, 2 | 0.37 | –6.71 | 0.46 | –4.66 | 0.78 | | –4.94 |
| Layer-3 | Sr 1 | 0, 0, 3/2 | –2.31 | –1.58 | –1.94 | 1.40 | –2.23 | | –0.94 |
|  | Sr 2 | 1, 0, 3/2 | 1.50 | 0.41 | 1.84 | 3.32 | 1.67 | | 2.18 |
|  | O 9 | 1/2, 1/2, 3/2 | 6.06 | 0.60 | 6.18 | 4.01 | 6.25 | | 2.51 |
|  | O 10 | 3/2, 1/2, 3/2 | –5.97 | –1.97 | –5.00 | 0.78 | –5.02 | | –1.09 |
| Layer-4 | Ti 5 | 1/2, 1/2, 1 | –0.01 | –0.88 | 0.42 | 2.92 | –1.20 | | –0.80 |
|  | Ti 6 | 3/2, 1/2, 1 | –0.84 | –0.55 | –0.46 | 3.99 | –2.24 | | 0.77 |
|  | O 11 | 0, 1/2, 1 | –0.10 | 3.26 | 0.30 | 6.84 | 1.04 | | 4.10 |
|  | O 12 | 1, 1/2, 1 | –0.53 | –5.13 | –0.18 | 0.00 | 0.85 | | 1.83 |
|  | O 13 | 1/2, 0, 1 | –0.37 | 0.33 | 0.00 | 4.76 | 0.36 | | –4.00 |
|  | O 14 | 3/2, 0, 1 | –0.27 | –1.40 | 0.10 | 2.46 | 0.78 | | –0.94 |
| Layer-5 | Sr 1 | 0, 0, 1/2 | –0.52 | –0.31 | N/A | N/A | N/A | | N/A |
|  | Sr 2 | 1, 0, 1/2 | 0.06 | –0.58 | N/A | N/A | N/A | | N/A |
|  | O 9 | 1/2, 1/2, 1/2 | –3.13 | –0.07 | N/A | N/A | N/A | | N/A |
|  | O 10 | 3/2, 1/2, 1/2 | 2.57 | –0.67 | N/A | N/A | N/A | | N/A |

Ref 3: N. Erdman *et al*.,^[4]^

Ref 4: T. Matsuda *et al*.,^[5]^

**Note S3. Choosing (2**×**1) surface unit cell for investigating reconstructed surfaces**

There are reports of the reconstructed STO (001) surface with many different periodicities including (1×1)^[6–8]^, (2×1)^[4,5,7,9–13]^, (2×2)^[7,9,14–16]^, c(4×2)^[10,11,13,16–18]^, c(4×4)^[10,16,19]^, c(6×2)^[11–13,20,21]^, (√5×√5-R26.6°)^[16,18,19,22–24]^, and (√13×√13-R33.7°)^[16,25,26]^ reconstructions. We investigate the reconstructed surface using (2×1) surface unit cell, which can be used to describe (1×1) and (2×1) reconstructions. One of the benefits of choosing the (2×1) unit cell is that the information obtained from the (2×1) investigation can be used to understand and predict the stable structures with different periodicities. For example, a (2×1) dimer model is used to understand the p(2×2) and c(4×2) reconstructions in Si (001) surface^[27]^. Also, STO (001) surface of TiO_2_-addlayer with (√10×√10-R18.4°) reconstruction has been predicted using TiO_5_ truncated octahedral units found in the (2×1) reconstructed surface^[28]^. Likewise, the information from the newly found SrTi_2_O_3_ surface with (2×1) unit cell, such as there should be edge-sharing TiO_5_ and TiO_4_ octahedra on the surface with Sr atoms on A site, can be used to predict other reconstruction with different periodicity. A SrTi_2_O_3_ surface with (2×2) reconstruction is found using the information (Figure S2).

**
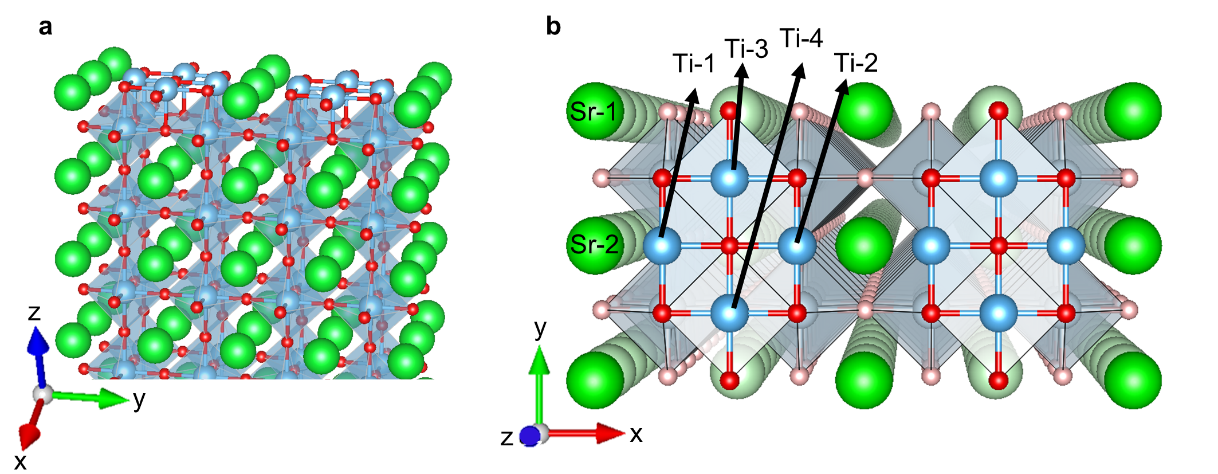
**

**Figure S2.** Atomic structures of a predicted SrTi_2_O_3_ (001) surface with (2×2) reconstruction. A (2×2) SrTi_2_O_3_ surface with edge-sharing TiO_5_ (Ti-3 and Ti-4) and TiO_4_ (Ti-1 and Ti-2) octahedra and two Sr atoms on the A sites are predicted using the information obtained from the SrTi_2_O_3_ surface with (2×1) unit cell. Side view (a) and top view (b). The *z*-direction is normal to the surface. The green, cyan, and red spheres represent the Sr, Ti, and O atoms, respectively, and the Ti-centered octahedrons are shaded in cyan. In (b), the atoms not on the surface are blurred in order to focus on the surface structure.

**Note S4. Constructing the candidate surface structures and the geometric screening**

We constructed the candidate surface structures on both TiO_2_ (Figure S3a) and SrO sub-surface (Figure S3b). Considering TiO_2_ sub-surface, there are two A sites, two O sites, and four octahedral interstitial sites (OIS) in the (2×1) surface unit cell (Figure S3a). Since there are 4^8^ = 65536 configurations to occupy these eight sites with four possibilities (Sr, Ti, O, and vacancy), it is not practically achievable to investigate all the configurations using DFT calculations. We remove unreasonable surface structures which do not satisfy any of the following conditions: (i) The OIS should not be occupied by Sr or O atoms. (ii) 12 coordinated A-site should not be occupied by Ti atom. (iii) O-site should not be occupied by Sr or Ti atoms. (iv) Ti atoms should not be sandwiched by adjacent Sr atoms. (v) The number of O atoms should not be less than that of the Ti atom. (vi) the surface should contain at least two species of Sr, Ti, and O. Already-known SrO-SL and TiO_2_-DL^[4,5,12,13]^ surfaces on TiO_2_ sub-surface with and without atomic vacancies are also excluded. We found that 14 candidate surface structures with six different stoichiometry fulfill these conditions on the TiO_2_ sub-surface (see Table S4).

Considering SrO sub-surface, there are two B sites, four O sites, and two A_top_ sites on top of the A site of the sub-surface SrO layer. We apply the same conditions (i)-(vi) as the TiO_2_ sub-surface case to eliminate unreasonable candidate structures. Additionally, we use one more condition for the B site: (vii) B site should not be occupied by O atom. Already-known TiO_2_-SL surfaces on SrO sub-surface with and without atomic vacancy are excluded from the candidate structures. Finally, we get 12 structures with four different surface stoichiometry, as shown in Table S4.


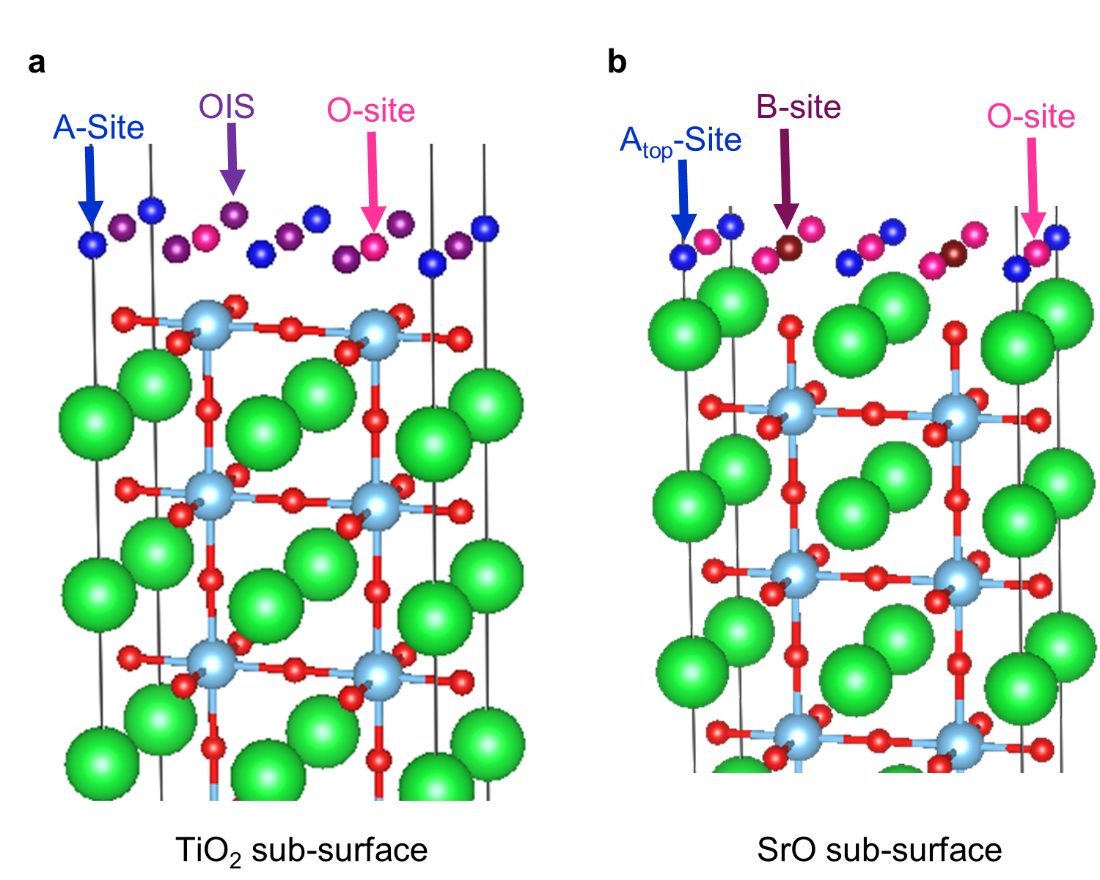


**Figure S3.** Ideal positions of the candidate surface structure. The ideal positions of the candidate surface structures with TiO_2_ sub-surface (a) and SrO sub-surface (b) are shown. A, B, and O stand for the perovskite’s A, B, and O sites, respectively. OIS indicates the octahedral interstitial site, and A_top_ indicates the position on top of the sub-surface A site. The green, cyan, and red spheres represent Sr, Ti, and O atoms, respectively.

**Table S4.** Candidate surface structures. The surface stoichiometry of the candidate surface structures and the number of structures for the surface stoichiometry are presented for both TiO_2_ sub-surface and SrO sub-surface cases.

| Sub-surface | Surface  stoichiometry | | Number of structures | |
| --- | --- | --- | --- | --- |
| TiO_2_ | SrTiO_3_ | | 2 | |
|  | SrTi_2_O_3_ | | 2 | |
|  | SrTiO_2_ | | 2 | |
|  | SrTi_2_O_2_ | | 5 | |
|  | SrTi_3_O_3_ | | 1 | |
|  | SrTi_3_O_2_ | | 2 | |
| SrO | Sr_2_O_2_ | | 2 | |
|  | SrTiO_3_ | | 3 | |
|  | SrTiO_2_ | | 6 | |
|  | SrTi_2_O_4_ | | 1 | |
| Total | | 26 | |  |

**Note S5. Examining the stability of the candidate surfaces by DFT total energy**

After the geometric screening of the candidate surface structures, we get 26 structures with ten different surface stoichiometry. We relax the atomic positions of these candidate structures by minimizing the DFT total energy. For each surface stoichiometry, we choose the lowest energy structure of the surface stoichiometry. As a result, the number of candidate surface structures is reduced to 10 (Description of these structures is provided in Figure S4, and Table S5). We estimate the stability of the ten candidate surface structures as well as the three known surfaces by calculating the surface formation energy per unit area *Ω_j_*(𝛥𝜇_Sr_,𝛥𝜇_O_) at 0 K without phonon correction for the surfaces *j (j* = SrO-SL, TiO_2_-SL, TiO_2_-DL, and the ten candidate structures), as a function of the chemical potentials of Sr (𝛥𝜇_Sr_) and O (𝛥𝜇_O_), by calculating the DFT total energy. To start with, we define the surface formation energy at 0 K, *E_f_*, as

*E_f_*(𝜇_x_) = 1/2 [*E*^DFT^_slab_ – 𝛴_X_ *n*_X_ 𝜇_x_] (1),

where *E*^DFT^_slab_ is the DFT total energy of the slab containing two identical surfaces on both sides, *n*_X_ is the number of atoms in the slab, 𝜇_x_ is the chemical potential of species X (X=Sr, Ti, and O). We use the equilibrium condition, ε_STO_ = 𝜇_Sr_ + 𝜇_Ti_ + 3𝜇_O_, where ε_STO_ is the DFT total energy of the bulk STO per unit cell, which is defined as ε_STO_ = *E*^DFT^_STO_ / *N*_cell_, where *N*_cell_ is the number of unit cells in the calculation. With the equilibrium condition, the Eqn. (1) becomes

*E_f_*(𝜇_Sr_,𝜇_O_) = 1/2 [*E*^DFT^_slab_ –*n*_Ti_ ε_STO_ – (*n*_Sr_ – *n*_Ti_)𝜇_Sr_ – (*n*_O_ – 3*n*_Ti_) 𝜇_O_] (2).

By introducing new variables, 𝛥𝜇_X_ = 𝜇_X_ – 𝜇^0^_X_, we get the final expression for *Ω^j^*,

*Ω^j^*(𝛥𝜇_Sr_,𝛥𝜇_O_) = *E_f_*(𝛥𝜇_Sr_,𝛥𝜇_O_)/*A* = 1/(2*A*)[*E*^DFT^_slab_ – *n*_Ti_ ε_STO_ – (*n*_Sr_ – *n*_Ti_)(𝛥𝜇_Sr_ + 𝜇^0^_Sr_) – (*n*_O_ – 3*n*_Ti_)(𝛥𝜇_O_ + 𝜇^0^_O_)] (3),

where 𝜇^0^_Sr_ and 𝜇^0^_O_ are the chemical potentials of Sr bulk and O_2_ molecule, respectively, and *A* is the surface area. Here, 𝜇^0^_X_ is defined as 𝜇^0^_X_ = *E*^DFT^_X_ /*N*_X_, where *N*_X_ is the number of X atoms in the unit cell. To obtain the thermodynamically allowed conditions for perovskite STO, we use five boundary conditions (BCs): (i) 𝛥𝜇_Sr_ ⩽0 for non-precipitation of Sr metal, (ii) 𝛥𝜇_O_ ⩽0 for non-evaporation of O_2_ molecule, (iii) 𝛥𝜇_Sr_ + 3𝛥𝜇_O_ ⩾ 𝛥*E*_STO_ for non-precipitation of Ti metal, (iv) 𝛥𝜇_Sr_ + 𝛥𝜇_O_ ⩾ 𝛥ε_STO_ – 𝛥ε_TiO2_ for non-precipitation of SrO bulk, and (v) 𝛥𝜇_Sr_ + 𝛥𝜇_O_ ⩽ 𝛥ε_SrO_ for non-precipitation of TiO_2_ bulk, where 𝛥ε_STO_ = ε_STO_ – 𝜇^0^_Sr_ – 𝜇^0^_Ti_ – 3𝜇^0^_O_, 𝛥ε_SrO_ = ε_SrO_ – 𝜇^0^_Sr_ – 𝜇^0^_O_, 𝛥ε_TiO2_ = ε_TiO2_ – 𝜇^0^_Ti_ – 2𝜇^0^_O_, and ε_TiO2_ and ε_SrO_ are the DFT total energy per unit cell of TiO_2_ and SrO bulk, respectively. The energy comparison among the known surfaces and the candidate surfaces is presented in Figure S5, showing the most stable surface structure as a function of 𝛥𝜇_Sr_ and 𝛥𝜇_O_. The thermodynamically allowed region of the bulk STO is represented by lines 1-5 corresponding to the BCs (i)-(v), respectively. Besides the already-known SrO-SL and TiO_2_-SL surfaces, only SrTi_2_O_3_ surface appear in the allowed region. The relaxed atomic positions of SrTi_2_O_3_ surface are presented in Table S6.

**
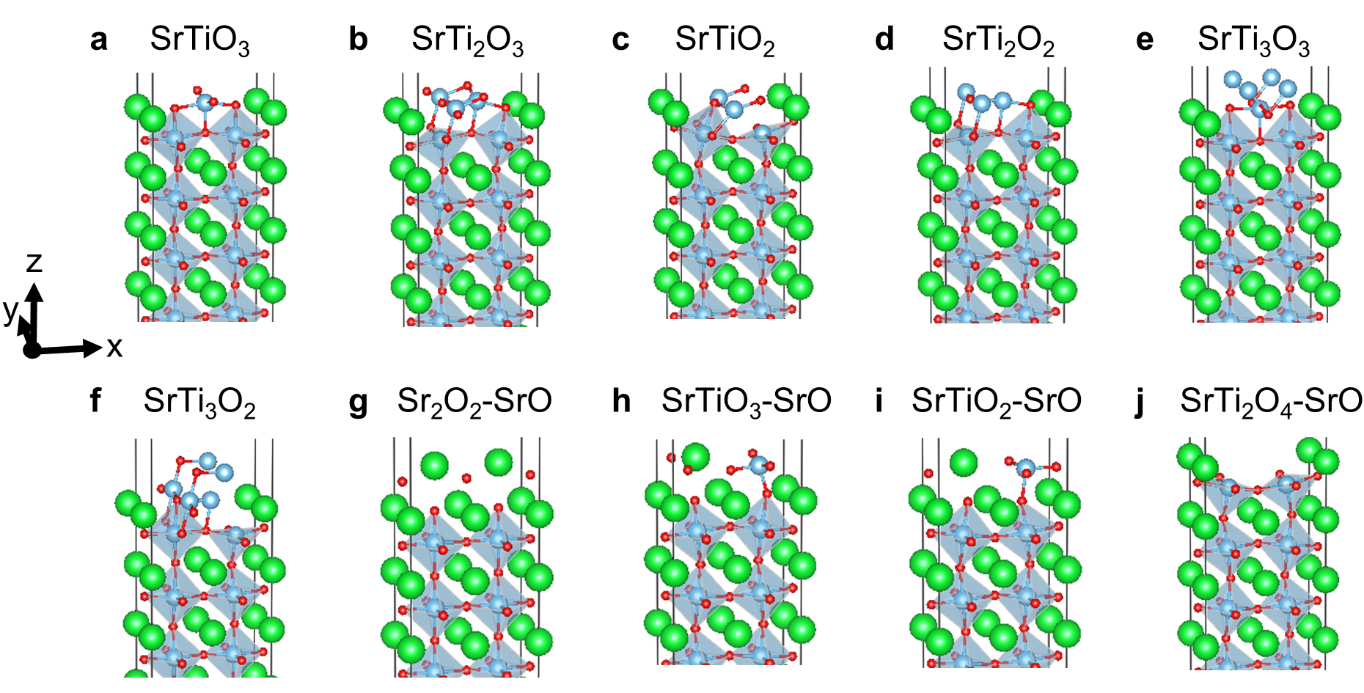
**

**Figure S4.** Relaxed structures of the candidate structures. For each stoichiometry, the relaxed atomic structure with the lowest energy is shown here**.** The green, cyan, and red spheres represent Sr, Ti, and O atoms, respectively, and the Ti-centered octahedrons are shaded in cyan.

**Table S5.** Relaxed atomic structures of the candidate structures. The displacements from the ideal atomic positions of the ten candidate surfaces, which have the lowest DFT energy among the structures with the same stoichiometry, are presented. The *z*-position of the atoms on the 6^th^ layer from the surface ideal atomic position is assigned to 0. The atomic displacements of the surface atoms are presented here. Units of Δ*x* and Δ*z* are in percent of the lattice constant *a*. Positive (negative) value of Δ*z* is for the displacement in the direction outward (inward) from the surface. For Δ*x*, see Figure S4 for the convention of the in-plane axes.

| Candidate surface structures | Atoms | Ideal atomic position  *x*, *y*, *z*  *[a]* | Atomic displacements [respect to the lattice constant(*a*) in %] | |
| --- | --- | --- | --- | --- |
|  |  |  | Δ*x* | Δ*z* |
| SrTiO_3_ | Sr 1 | 0, 0, 5/2 | 0 | –5.94 |
|  | Ti 1 | 1, 1/2, 5/2 | 0 | 5.28 |
|  | O 1 | 1/2, 1/2, 5/2 | –0.60 | –1.35 |
|  | O 2 | 1, 0, 5/2 | 0 | 16.66 |
|  | O 3 | 3/2, 1/2, 5/2 | 0.60 | –1.35 |
| SrTi_2_O_3_ | Sr 1 | 0, 0, 5/2 | –13.26 | 5.05 |
|  | Ti 1 | 1/2, 0, 5/2 | 10.22 | –8.81 |
|  | Ti 2 | 1, 1/2, 5/2 | 8.66 | –12.34 |
|  | O 1 | 1, 0, 5/2 | 5.22 | –27.99 |
|  | O 2 | 3/2, 1/2, 5/2 | 6.82 | 1.71 |
|  | O 3 | 1/2, 1/2, 5/2 | 0.21 | –10.78 |
| SrTiO_2_ | Sr 1 | 0, 0, 5/2 | 8.18 | –1.51 |
|  | Ti 1 | 1, 0, 5/2 | –11.40 | 0.22 |
|  | O 1 | 1/2, 1/2, 5/2 | –0.60 | –1.35 |
|  | O 2 | 3/2, 0, 5/2 | –17.31 | 13.21 |
| SrTi_2_O_2_ | Sr 1 | 0, 0, 5/2 | –11.15 | –6.63 |
|  | Ti 1 | 1/2, 0, 5/2 | 10.83 | 17.66 |
|  | Ti 2 | 1, 1/2, 5/2 | –39.17 | 17.66 |
|  | O 1 | 1/2, 1/2, 5/2 | 4.90 | 15.58 |
|  | O 2 | 3/2, 1/2, 5/2 | 6.27 | 0.37 |
| SrTi_3_O_3_ | Sr 1 | 0, 0, 5/2 | 0 | –5.58 |
|  | Ti 1 | 1/2,0,5/2 | 17.47 | 44.90 |
|  | Ti 2 | 1, 1/2, 5/2 | 0 | 3.67 |
|  | Ti 3 | 3/2, 0, 5/2 | –17.47 | 44.90 |
|  | O 1 | 1/2, 1/2, 5/2 | –0.28 | 7.38 |
|  | O 2 | 1, 0, 5/2 | 0 | 4.09 |
|  | O 3 | 3/2, 1/2, 5/2 | 0.28 | 7.38 |
| SrTi_3_O_2_ | Sr 1 | 0, 0, 5/2 | –13.01 | 0.02 |
|  | Ti 1 | 1/2, 0, 5/2 | 12.63 | 19.35 |
|  | T 2 | 1, 1/2, 5/2 | 11.24 | 8.38 |
|  | T 3 | 3/2, 0, 5/2 | –29.80 | 62.10 |
|  | O 1 | 1/2, 1/2, 5/2 | 5.83 | 10.68 |
|  | O 2 | 1, 0, 5/2 | –25.66 | 65.26 |
| Sr_2_O_2_–SrO | Sr 1 | 1/2, 1/2, 5/2 | 0 | 18.44 |
|  | Sr 2 | 3/2, 1/2, 5/2 | 0 | 18.44 |
|  | O 1 | 0, 1/2, 5/2 | 0 | –3.33 |
|  | O 2 | 1, 1/2, 5/2 | 0 | –3.33 |
| SrTiO_3_–SrO | Sr 1 | 1/2, 1/2, 5/2 | –1.97 | 15.49 |
|  | Ti 1 | 3/2, 1/2, 5/2 | –2.84 | –2.62 |
|  | O 1 | 1/2, 0, 5/2 | –27.13 | 6.09 |
|  | O 2 | 1, 1/2, 5/2 | 2.71 | –7.78 |
|  | O 3 | 3/2, 0, 5/2 | 0.68 | 5.83 |
| SrTiO_2_–SrO | Sr 1 | 1/2, 1/2, 5/2 | 1.61 | 12.03 |
|  | Ti 1 | 3/2, 1/2, 5/2 | –1.04 | –1.89 |
|  | O 1 | 0, 1/2, 5/2 | –3.02 | –1.79 |
|  | O 2 | 3/2, 0, 5/2 | –14.01 | 0.63 |
| SrTi_2_O_4_–SrO | Sr 1 | 0, 0, 5/2 | 0.00 | 42.57 |
|  | Ti 1 | 1/2, 1/2, 5/2 | 1.93 | –1.79 |
|  | Ti 2 | 3/2, 1/2, 5/2 | –1.93 | –1.79 |
|  | O 1 | 0, 1/2, 5/2 | 0.00 | 9.85 |
|  | O 2 | 1/2, 0, 5/2 | –0.43 | 5.69 |
|  | O 3 | 1, 1/2, 5/2 | 0.00 | –8.35 |
|  | O 4 | 3/2, 0, 5/2 | 0.43 | 5.69 |


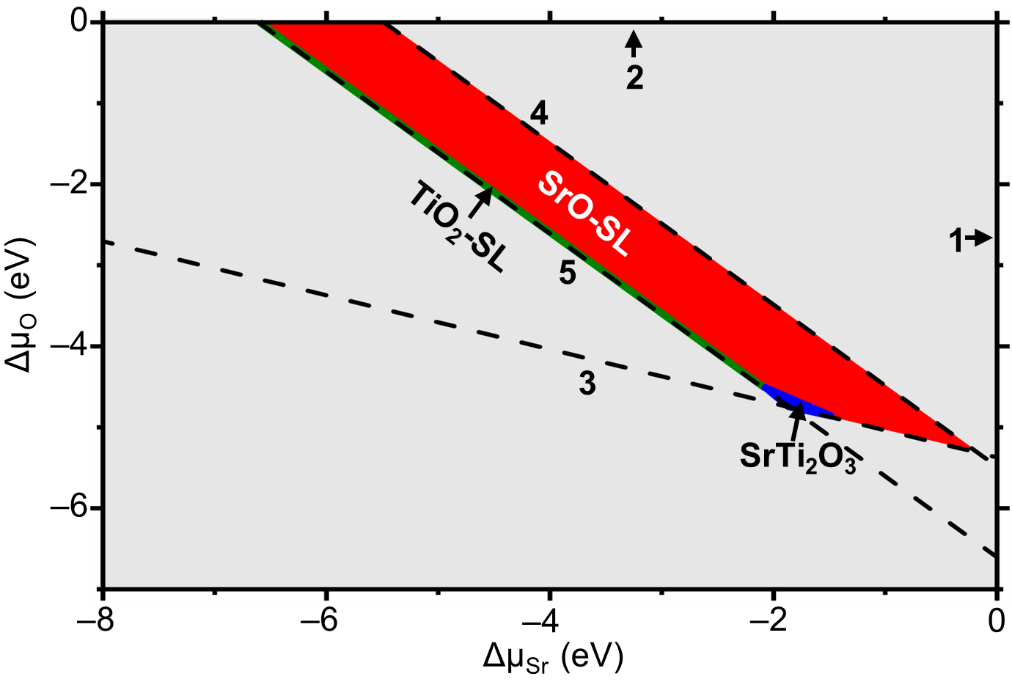


**Figure S5.** Scrutinizing candidate surface structures. Energy comparison of candidate surfaces and the three known surfaces is presented showing the most stable surface structure as a function of 𝛥𝜇_Sr_ and 𝛥𝜇_O_ within the perovskite STO allowed region. The allowed region of the bulk perovskite STO is represented by lines 1-5 corresponding to BCs (i)-(v), respectively. BC (i): the non-precipitation limit of Sr metal, BC (ii): the non-evaporation limit of O_2_ molecule and BC (iii): the non-precipitation limit of Ti metal, BC (iv): the non-precipitation limit of SrO bulk, and BC (v): the non-precipitation limit of TiO_2_ bulk.

**Table S6.** Relaxed atomic positions of the SrTi_2_O_3_ (2×1) surface structure. Calculated atomic displacements from the ideal positions of the SrTi_2_O_3_ surface are presented. The *z*-position of the atoms on the 6^th^ layer from the surface ideal atomic position is assigned to 0. Positive (negative) value of Δ*z* is for the displacements in the direction outward (inward) from the surface. For Δ*x*, see Figure 1 in the main article for the convention of the in-plane axes.

| Layers | Atoms | Symmetric atomic position  *x*, *y*, *z*  [*a*] | Atomic displacements [respect to the lattice constant(*a*) in %] | |
| --- | --- | --- | --- | --- |
|  |  |  | Δ*x* | Δ*z* |
| Layer-1  (Surface) | Sr 1 | 0, 0, 5/2 | –13.26 | 5.05 |
|  | Ti 1 | 1/2, 0, 5/2 | 10.22 | –8.81 |
|  | Ti 2 | 1, 1/2, 5/2 | 8.66 | –12.34 |
|  | O 1 | 1, 0, 5/2 | 5.22 | –27.99 |
|  | O 2 | 3/2, 1/2, 5/2 | 6.82 | 1.71 |
|  | O 3 | 1/2, 1/2, 5/2 | 0.21 | –10.78 |
| Layer-2  (Sub-Surface) | Ti 3 | 1/2, 1/2, 2 | –1.12 | –1.32 |
|  | Ti 4 | 3/2, 1/2, 2 | –1.82 | 2.82 |
|  | O 4 | 0, 1/2, 2 | –1.75 | 4.55 |
|  | O 5 | 1, 1/2, 2 | –0.65 | –8.27 |
|  | O 6 | 1/2, 0, 2 | –6.56 | –11.55 |
|  | O 7 | 3/2, 0, 2 | –0.59 | 4.28 |
| Layer-3 | Sr 2 | 0, 0, 3/2 | –0.52 | 0.84 |
|  | Sr 3 | 1, 0, 3/2 | 0.19 | 2.94 |
|  | O 8 | 1/2, 1/2, 3/2 | 5.14 | 0.13 |
|  | O 9 | 3/2, 1/2, 3/2 | –6.42 | 1.25 |
| Layer-4 | Ti 5 | 1/2, 1/2, 1 | –0.09 | –0.28 |
|  | Ti 6 | 3/2, 1/2, 1 | –0.34 | 1.41 |
|  | O 10 | 0, 1/2, 1 | –0.45 | –3.98 |
|  | O 11 | 1, 1/2, 1 | –0.28 | 4.53 |
|  | O 12 | 1/2, 0, 1 | –0.48 | 0.23 |
|  | O 13 | 3/2, 0, 1 | –0.25 | 0.34 |
| Layer-5 | Sr 4 | 0, 0, 1/2 | 0.29 | 0.70 |
|  | Sr 5 | 1, 0, 1/2 | –0.75 | 0.95 |
|  | O 14 | 1/2, 1/2, 1/2 | –3.05 | 0.10 |
|  | O 15 | 3/2, 1/2, 1/2 | 2.79 | 0.29 |

**Note S6. The phonon density of states, band structures and thermal response.**

Non-analytical term correction is applied to the dynamical matrix of all the materials investigated to treat the long-range interaction of macroscopic electric field^[29,30]^. The calculated phonon bands with and without non-analytical term correction (NAC), Phonon Density of States with and without NAC, Free energy with and without NAC, and contribution of vibrational energy and entropy terms to the free energy with NAC correction of STO bulk are presented in Figure S6a-S6d. The calculated phonon bands for STO bulk with and without Non-analytical term correction (NAC), Phonon Density of States with and without NAC, Free energy with and without NAC, and contribution of vibrational energy and entropy terms to the free energy with NAC correction for each species (Sr, Ti, SrO, and TiO_2_ bulk) and four surfaces (SrO-SL, TiO_2_-SL, TiO_2_-DL, and SrTi_2_O_3_) are presented in Figure S7a-S7p and S8a-S8p.


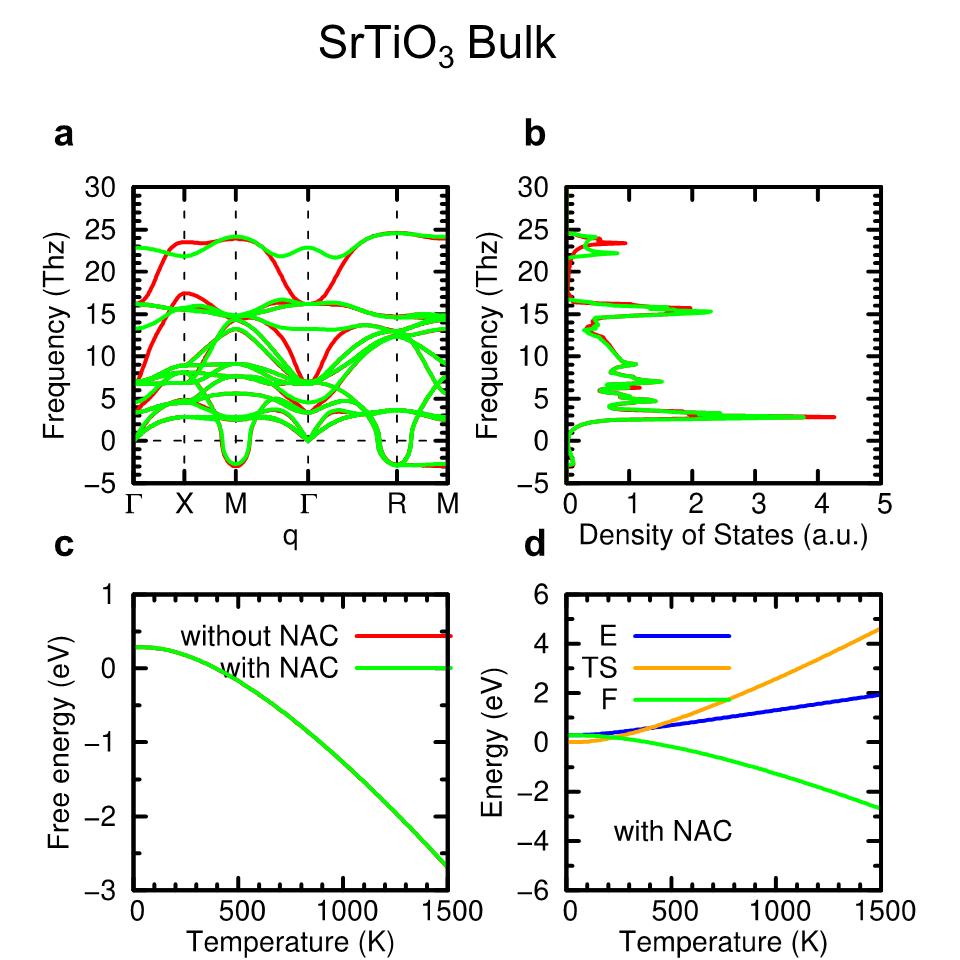


**Figure S6.** Phonon band structure, the density of states, and thermal response of STO bulk. the band structure with and without non-analytic term correction (NAC) (a), Phonon Density of States with and without NAC (b), Free energy correction with respect to temperature with and without NAC (c), and contribution of vibrational energy(E) and entropy terms(TS) to the free energy(F) with NAC correction (d) are presented in blue, orange and green color respectively.


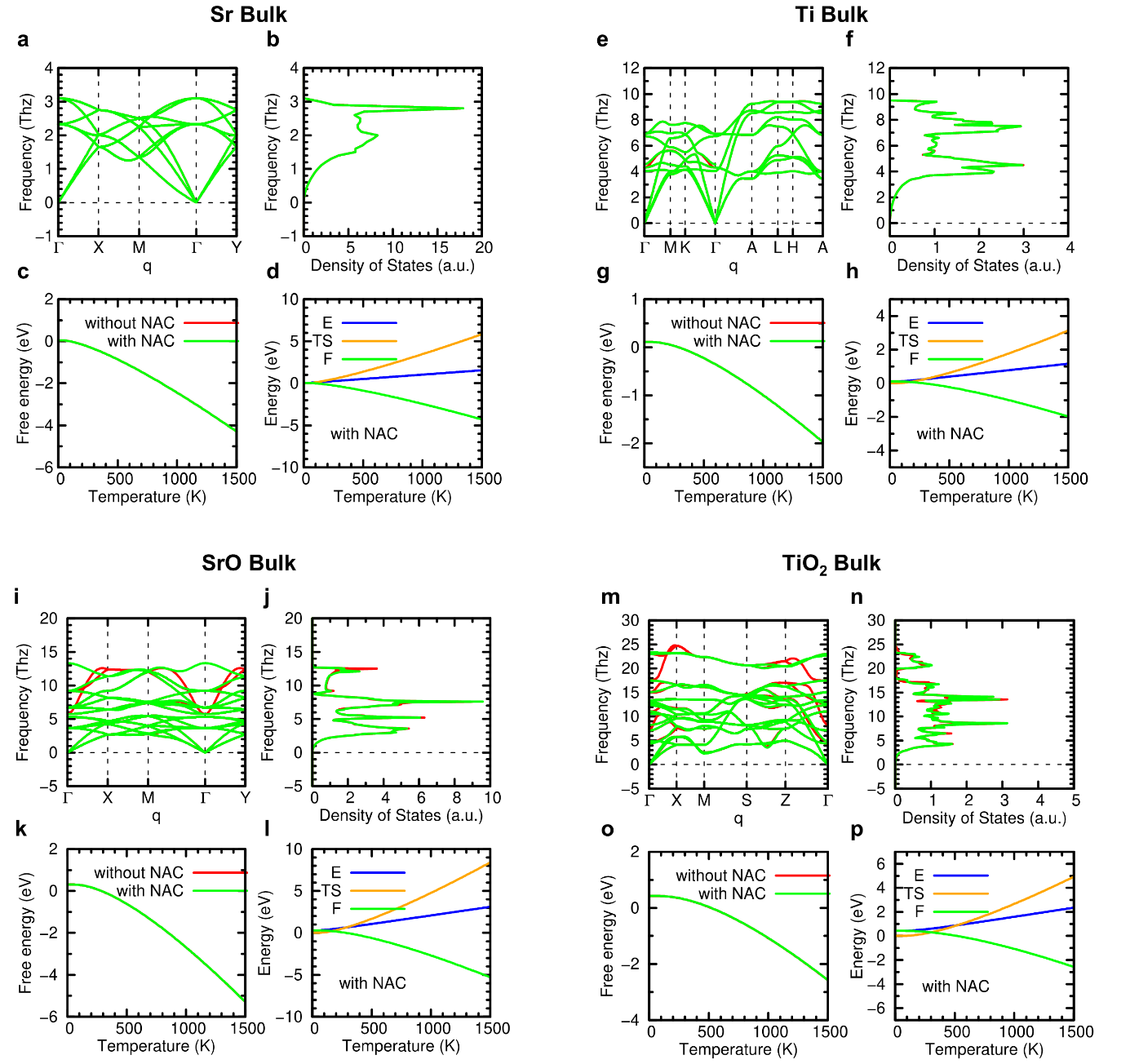


**Figure S7.** Phonon band structure, the density of states, and thermal response for (a-d) Sr, (e-h) Ti, (i-l) SrO bulk, and (m-p) TiO_2_ bulk. For each species, the band structure with and without non-analytic term correction (NAC) (a, e, i, and m), Phonon Density of States with and without NAC (b, f, j, and n), Free energy correction with respect to temperature with and without NAC (c, g, k, and o), and contribution of vibrational energy and entropy terms to the free energy with NAC correction (d, h, l, and p) are presented.


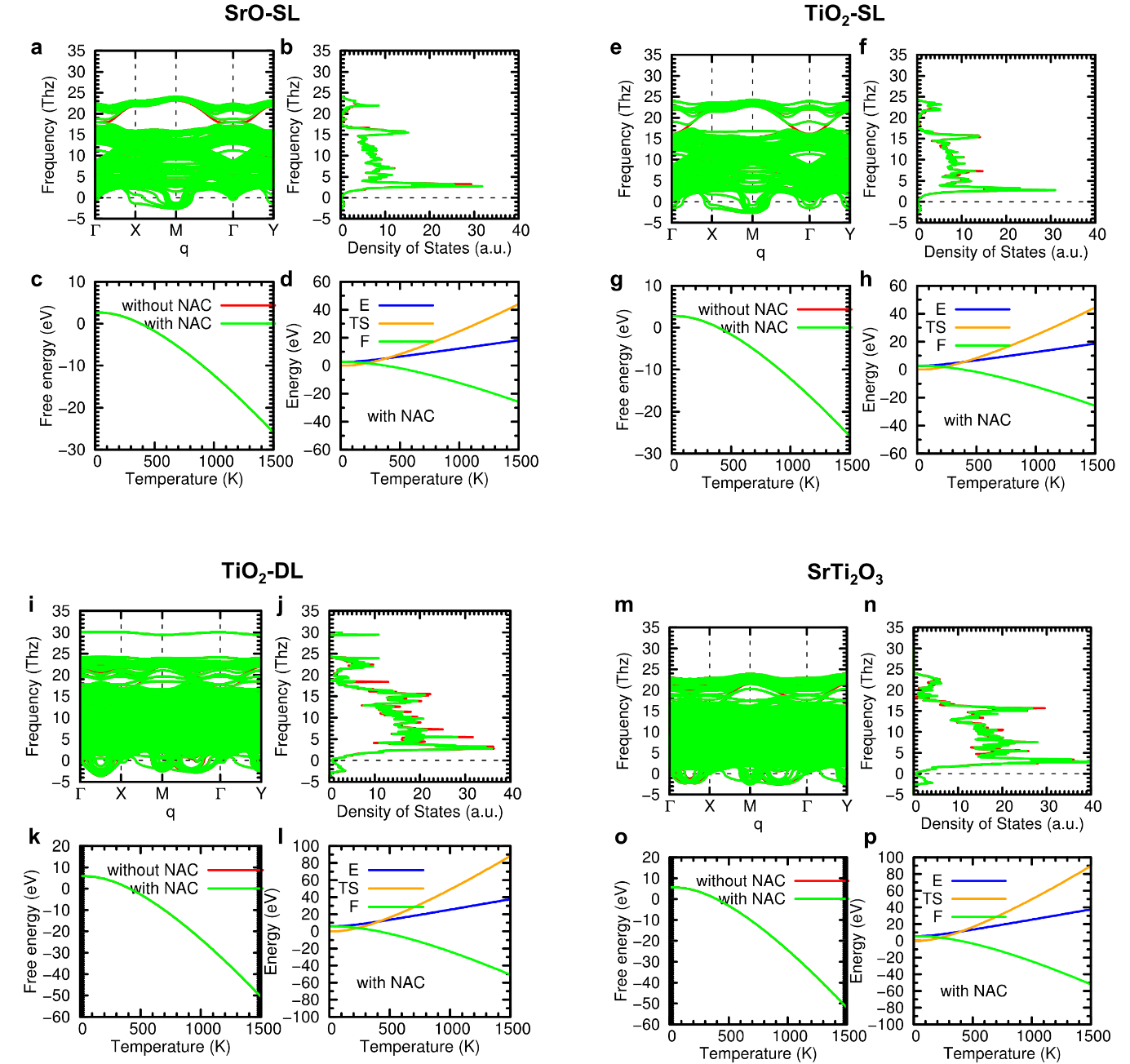


**Figure S8.** Phonon band structure, the density of states, and thermal response of four surfaces (a-d) SrO-SL, (e-h) TiO_2_-SL, (i-l) TiO_2_-DL, (m-p), and SrTi_2_O_3_. For each surface, the band structure with and without non-analytic term correction (NAC) (a, e, I, and m), Phonon Density of States with and without NAC (b, f, j, and n), Free energy correction with respect to temperature with and without NAC (c, g, k, and o), and contribution of vibrational energy and entropy terms to the free energy with NAC correction (d, h, l, and p) are presented. For SrO-SL and TiO_2_-SL the energy are for 1$\times$1 surface area, while for TiO_2_-DL and SrTi_2_O_3_ the energies for 2$\times$1 surface area.

**Note S7. Thermodynamic phase diagram (TPD) with respect to the oxygen partial**

**pressure at various temperatures**

The obtained temperature-dependent thermodynamic phase diagram (Figure 2 in the main article) is replotted as a function of the oxygen partial pressure, *P*_O2_, and the chemical potential of Sr, 𝛥𝜇_Sr_, using 𝛥𝜇_O_=*k*_B_*T ln*(*P*_O2_/*P*^0^)^[31,32]^, where *P*^0^ is the standard pressure (1 atm) (see Figure S9).


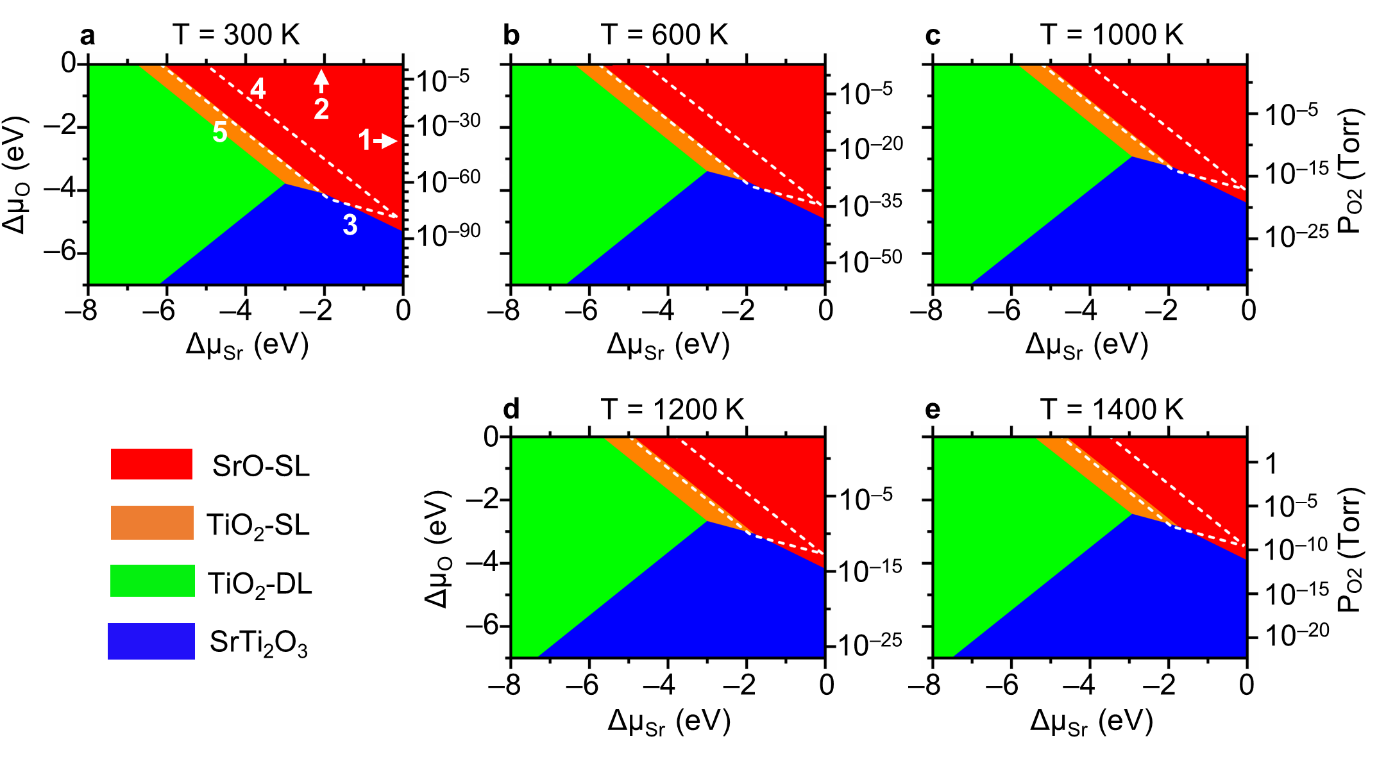


**Figure S9.** The obtained TPD of STO (001) surfaces at various temperatures with estimated *P*_O2_. The TPD shows the most stable surface as a function of 𝛥𝜇_Sr_ and 𝛥𝜇_O_ at 300 K (a), 600 K (b), 1000 K (c), 1200 K (d), and 1400 K (e). The allowed region of the bulk perovskite STO is represented by white dashed lines, where lines 1-5 correspond to BCs (i)-(v), respectively. BC (i): the non-precipitation limit of Sr metal, BC (ii): the non-evaporation limit of O_2_ molecule, BC (iii): the non-precipitation limit of Ti metal, BC (iv): the non-precipitation limit of SrO bulk, and BC (v): the non-precipitation limit of TiO_2_ bulk. On the right *y*-axis, the estimated *P*_O2_ is presented.

**Note S8. Obtaining oxygen chemical potential using empirical thermodynamic data**

In the main text, we obtained oxygen chemical potential at finite temperatures by phonon calculations. Here we use empirical thermodynamic data to obtain the chemical potential at finite temperature, and compare the results with what is presented in the main text.

The chemical potential of oxygen can be defined(33, 34) as,

𝜇^0^_O2_(*T*) = *E*^DFT^_O2_ + *E*^ZPE^_O2_(*T*) + 𝛥𝜇_O2_(*T, P_0_*)

= *E*^DFT^_O2_ + *E*^ZPE^_O2_(*T*) + 𝛥𝜇’_O2_(*T, P_0_*) + *K*Tln(P_O2_/P_0_)

Here, the DFT energy( *E*^DFT^_O2_) and zero-point vibrational energy(*E*^ZPE^_O2_(*T*) ) of O_2_ molecule are calculated at 0K. We use the reference pressure P_0_=1atm, which is commonly used. The oxygen chemical potential difference at 0K and finite temperature (*T*) (𝛥𝜇’_O2_(*T, P_0_*)) can be expressed in terms of enthalpy (𝛥H(T, P_0_)) and entropy (TS(T, P_0_)) of O_2_ at T and P_0_ with respect to the values at 0K, which has been obtained from JANAF thermodynamical tables(35).

𝛥𝜇’_O2_(*T, P_0_*) = 𝛥H(T, P_0_) – TS(T, P_0_)

The resulting thermodynamic phase transition using this definition of oxygen chemical potential is shown in Figure S10. This is a similar thermodynamic phase diagram as presented in Figure 2. The only difference between these two formalisms is that the thermodynamical allowed region and all four surface‘s stability regions move towards a lower oxygen chemical potential.

**
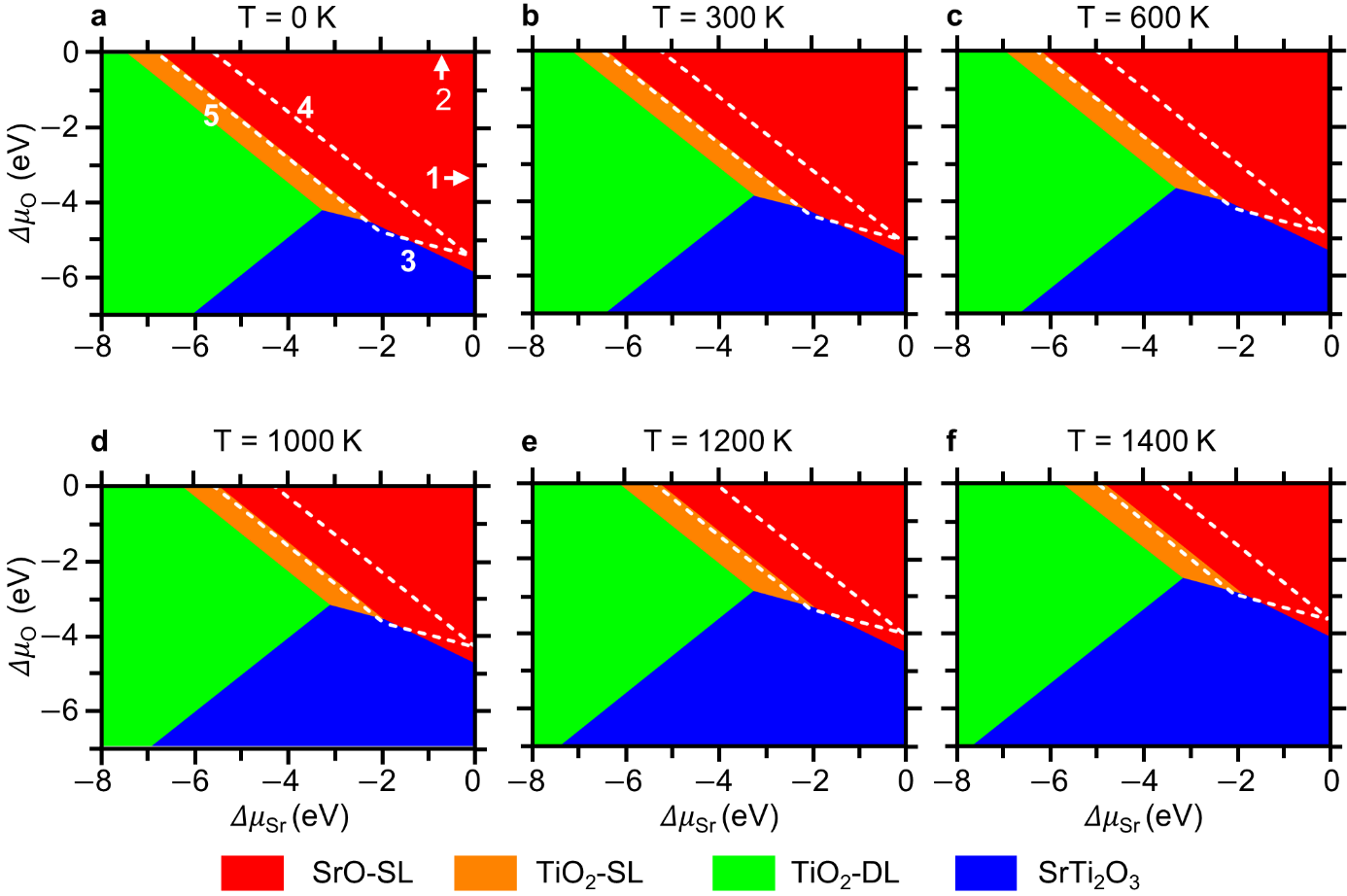
**

**Figure S10.** Temperature-dependent phase diagram (TPD) of STO (001) surface at various temperatures when oxygen chemical potential obtained from empirical thermodynamic data TPD shows the most stable surface as a function of 𝛥𝜇_Sr_ and 𝛥𝜇_O_ at temperature (a) 0 K, (b) 300 K, (c) 600 K, (d) 1000 K, (e) 1200 K, and (f) 1400 K. The allowed region of the bulk perovskite STO is represented by white dashed lines, where lines 1-5 correspond to BCs (i)-(v), respectively. BC (i): the non-precipitation limit of Sr metal, BC (ii): the non-evaporation limit of O_2_ molecule, BC (iii): the non-precipitation limit of Ti metal, BC (iv): the non-precipitation limit of SrO bulk, and BC (v): the non-precipitation limit of TiO_2_ bulk. The only difference between this and what is shown in Figure 2 (main text) is that the thermodynamical allowed region and all four surface‘s stability regions move towards a lower oxygen chemical potential.

**Note S9: Phonon energy and entropic contribution for the stability of STO (001) surfaces** In this section, we discuss a detailed comparison of the influence of phonon energy and entropic contributions on the surface stability of SrTiO_3_ (001). Figures S11-S13 show the contributions of phonon energy, entropic contribution, and a combination of phonon energy and entropic contributions, respectively.

Figure S11(a) shows phonon energy, including zero-point vibrational energy, with respect to temperature for four stable surfaces. It demonstrates that phonon energy is lower for SrO-SL and TiO_2_-SL surfaces compared to the reconstructed surfaces (SrTi_2_O_3_ and TiO_2_-DL), favoring the stability of single-layer surfaces. In Figure S11(b), we plotted the relative phonon energy change of the three surfaces to SrO-SL with temperature, setting the phonon energy of SrO-SL to zero as a reference. The relative phonon energy changes of the three surfaces (TiO_2_-SL, TiO_2_-DL, and SrTi_2_O_3_) are presented by E_vib_^TiO2-SL^ - E_vib_^SrO-SL^, E_vib_^TiO2-DL^ - E_vib_^SrO-SL^, and E_vib_^SrTi2O3^ - E_vib_^SrO-SL^, respectively. This comparison indicates that SrO-SL has the lowest phonon energy, followed by TiO_2_-SL, SrTi_2_O_3_, and TiO_2_-DL in sequence.

Figure S12(a) shows the entropic contribution (-TS) with respect to temperature for four stable surfaces. It demonstrates that the entropic contribution is most prominent for SrTi_2_O_3_ surface, favoring its thermodynamic stability. In Figure S12(b), we plotted the relative entropic term change of the three surfaces to SrO-SL with temperature, setting the entropic contribution of SrO-SL to zero as a reference. The relative entropic contribution changes of the three surfaces (TiO_2_-SL, TiO_2_-DL, and SrTi_2_O_3_) are presented by (-TS)^TiO2-SL^ – (-TS)^SrO-SL^; (-TS)^TiO2-DL^ – (-TS)^SrO-SL^; and (-TS)^SrTi2O3^ – (-TS) ^SrO-SL^_,_ respectively. The plot indicates that below 360K, SrO-SL surface has the most significant entropic contributions, while above 360K, SrTi_2_O_3_ surface shows the most prominent entropic contributions. Around 480K, the entropic contribution of TiO_2_-SL becomes more negative than that for SrO-SL but remains higher than that for SrTi_2_O_3_. At approximately 1150K, the entropic contribution of TiO_2_-DL surface becomes more prominent compared to SrO-SL surface but remains less significant than that for SrTi_2_O_3_ and TiO_2_-SL surfaces.

Note that the lower the phonon energy and the more negative the entropic contribution, the higher the stability of the surface, as both are added to the total energy to calculate the free energy, according to equation 2 in the main text.

*G*_slab_(*T*) = *E*^DFT^_slab_ + *E*^vib^_slab_(*T*) – *TS*^vib^_slab_(*T*)

We also plotted the combined effect of phonon energy and entropic contribution with temperature, as depicted in Figure S13. Figure S13(a) shows that the combined effect of phonon energy and entropy favors SrO-SL surface up to a certain temperature, above the temperature it favors the stability of SrTi_2_O_3_ surface. Figure S13(b) shows the relative combined effect of phonon energy and entropic contribution change of the three surfaces to SrO-SL with temperature, setting the combined effect of phonon energy and entropic contribution of SrO-SL to zero as a reference. The relative combined effect of phonon energy and entropic contribution change of the three surfaces (TiO_2_-SL, TiO_2_-DL, and SrTi_2_O_3_) are presented by (E_vib_-TS)^TiO2-SL^ – (E_vib_-TS)^SrO-SL^; (E_vib_-TS)^TiO2-DL^ – (E_vib_-TS)^SrO-SL^; and (E_vib_-TS)^SrTi2O3^ – (E_vib_-TS)^SrO-SL^ respectively. The plot indicates that up to 1100K, SrO-SL surface is favored by this combined effect, and above 1100K this combined effect of phonon energy and entropy favors SrTi_2_O_3_ surface.

To summarize, Phonon energy is the dominant factor over entropic contribution at lower temperatures, favoring the thermodynamic stability of SrO-SL and TiO_2_-SL surfaces. On the other hand, the entropic contribution becomes dominant over phonon energy at higher temperatures, favoring the stabilization of SrTi_2_O_3_ surface.


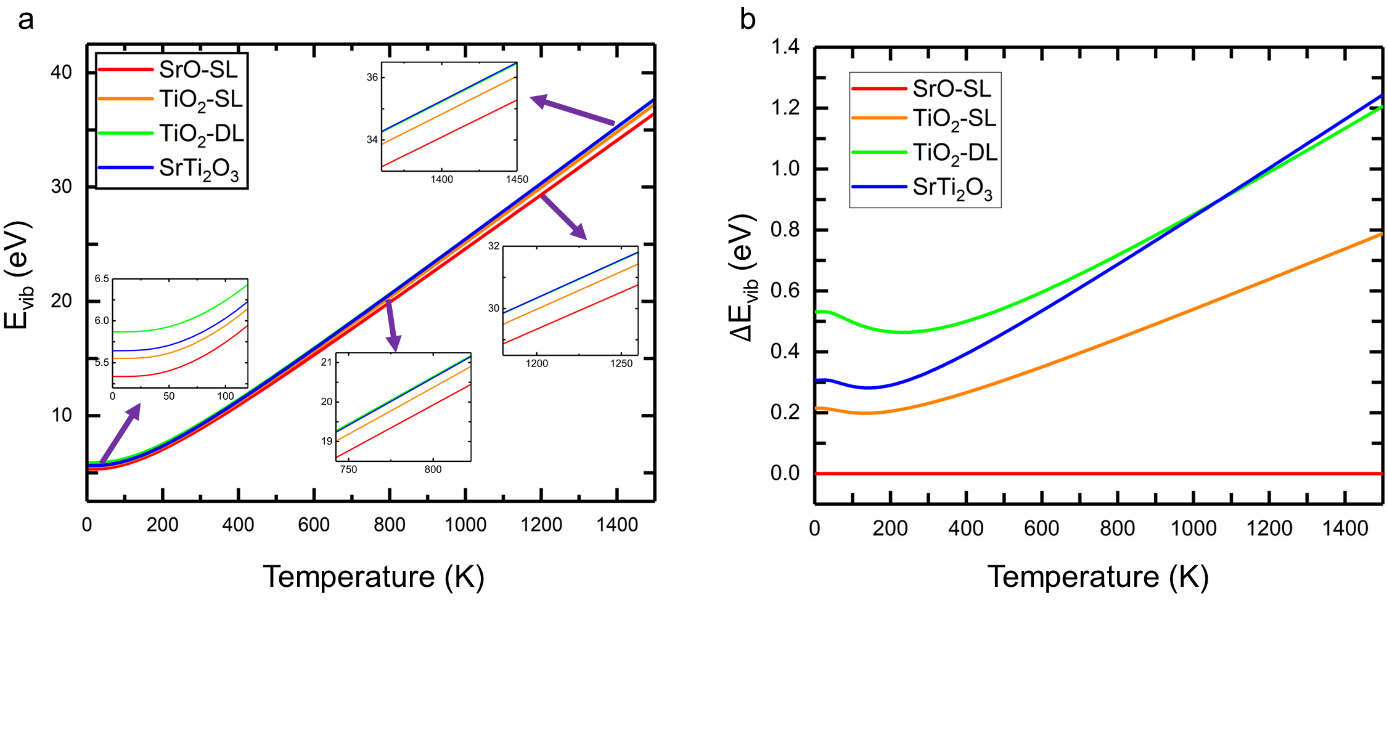


**Figure S11:** (a) Phonon energy, including zero-point vibrational energy (E_vib_), with respect to temperature for the four surfaces is shown. The insets display enlarged graphs within small temperature ranges for better visibility of trends at specific temperature intervals. The phonon energy is comparatively higher for TiO_2_-DL and SrTi_2_O_3_ surfaces, followed by TiO_2_-SL, and is lowest for SrO-SL. (b) To provide a clearer view, relative phonon energy changes of the three surfaces to SrO-SL with temperature are plotted, setting the phonon energy of SrO-SL to zero as a reference. The relative phonon energy changes of the three surfaces (TiO_2_-SL, TiO_2_-DL, and SrTi_2_O_3_) are presented by E_vib_^TiO2-SL^ - E_vib_^SrO-SL^, E_vib_^TiO2-DL^ - E_vib_^SrO-SL^, and E_vib_^SrTi2O3^ - E_vib_^SrO-SL^, respectively. All energies are shown for a (2×1) surface area for direct comparison.


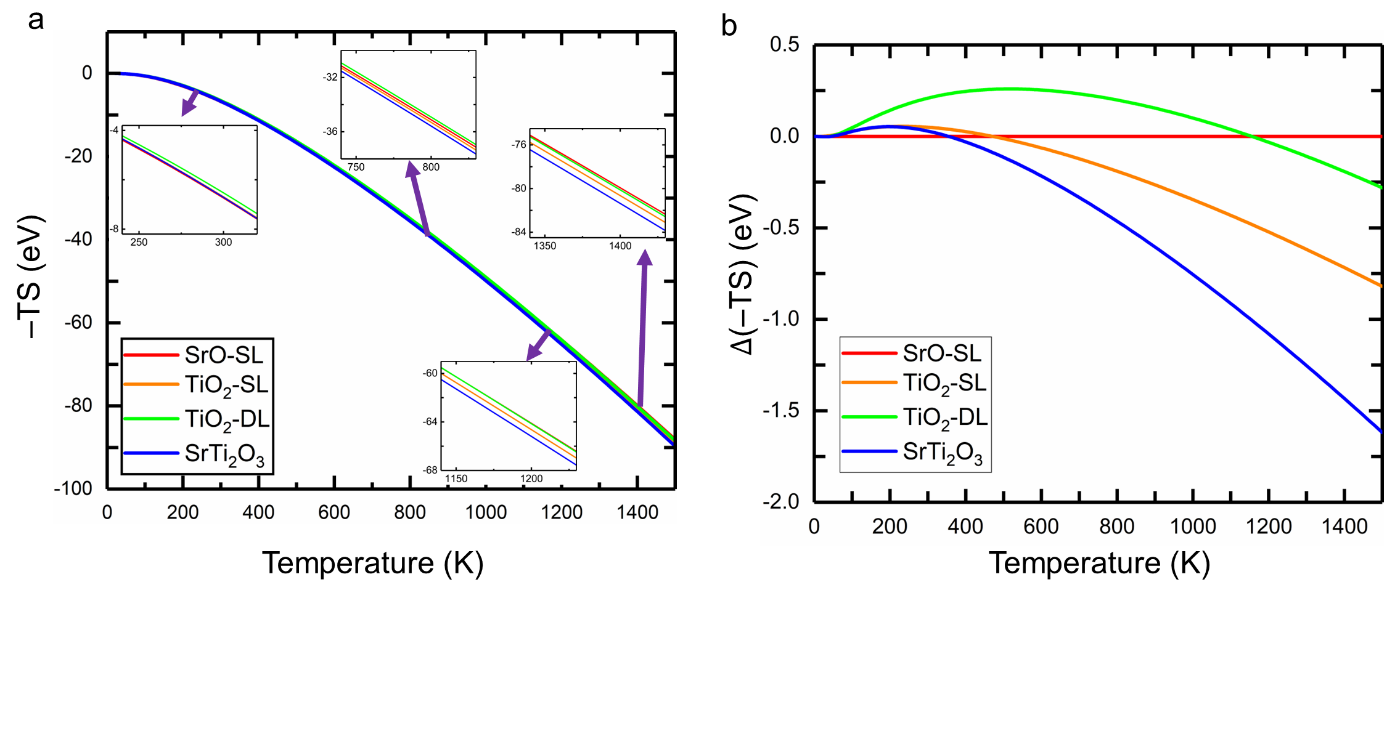


**Figure S12:** (a) Entropic contribution (-TS) with respect to temperature for the four surfaces is shown. The insets display enlarged graphs within smaller temperature ranges for better visibility of trends at specific temperature intervals. The entropic contribution is comparatively higher for SrTi_2_O_3_, followed by TiO_2_-SL and SrO-SL, and is lowest for TiO_2_-DL. With increasing temperature, the entropic contribution for SrTi_2_O_3_ becomes more prominent, and the contribution in TiO_2_-DL becomes larger than that in SrO-SL. (b) To provide a clearer view, relative entropic contribution changes of the three surfaces to SrO-SL with temperature are plotted, setting the entropic contribution of SrO-SL to zero as a reference. The relative entropic contribution changes of the three surfaces (TiO_2_-SL, TiO_2_-DL, and SrTi_2_O_3_) are presented by (-TS)^TiO2-SL^ – (-TS)^SrO-SL^; (-TS)^TiO2-DL^ – (-TS)^SrO-SL^; and (-TS)^SrTi2O3^ – (-TS)^SrO-SL^, respectively. All values are shown for a (2×1) surface area for direct comparison..


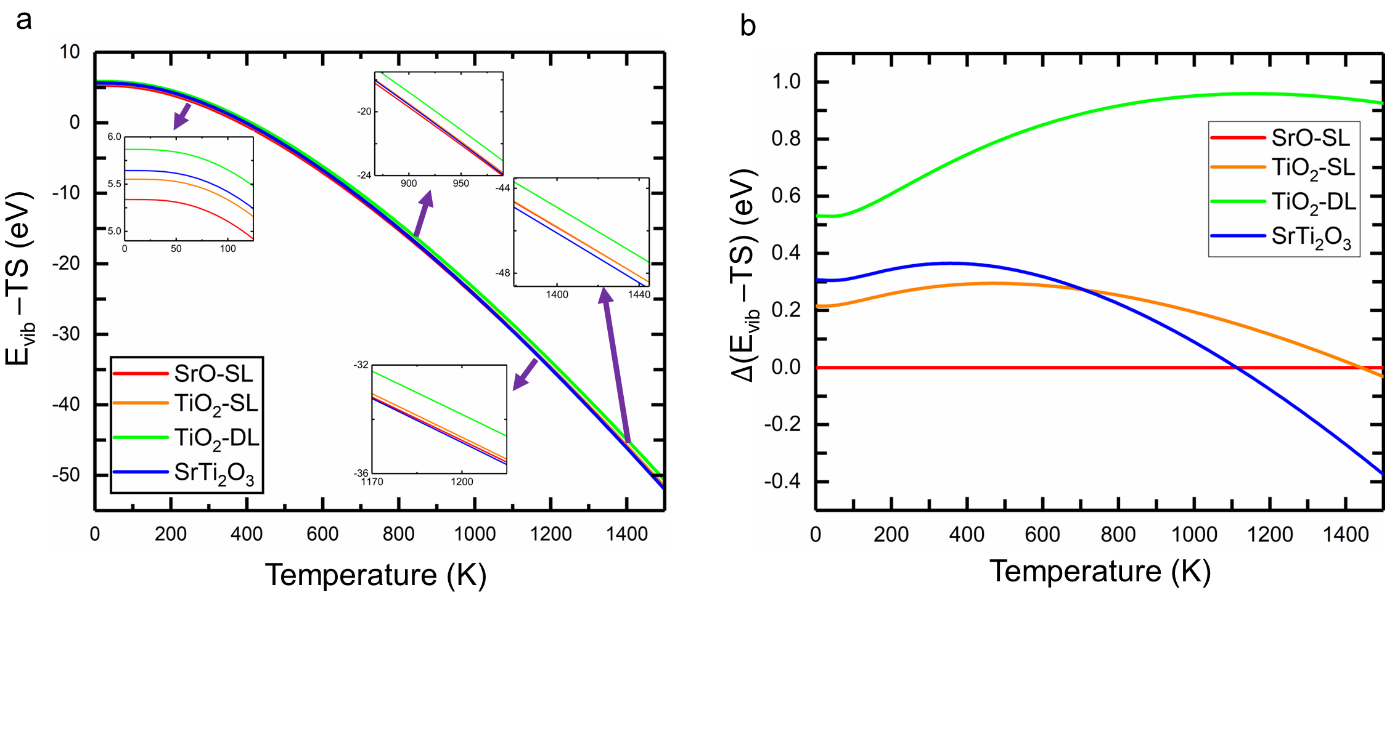


**Figure S13:** (a) Combined effect of phonon energy and entropic contribution (E_vib_ - TS) with respect to temperature for the four stable surfaces is shown. The insets display enlarged graphs within shorter temperature ranges for better visibility of trends at specific temperature intervals. At lower temperatures, the combined effect of phonon energy and entropy favors SrO-SL the most, followed by TiO_2_-SL, SrTi_2_O_3_, and TiO_2_-DL in sequence. With increasing temperature, this combined effect becomes more prominent for the SrTi_2_O_3_ surface, and at higher temperatures, the combined effect of phonon energy and entropy is most prominent for the SrTi_2_O_3_ surface. (b) For a clearer view, relative changes in the combined effect of phonon energy and entropic contribution of the three surfaces to SrO-SL with temperature are plotted, setting the combined effect of phonon energy and entropic contribution of SrO-SL to zero as a reference. The relative combined effect of phonon energy and entropic contribution changes of the three surfaces (TiO_2_-SL, TiO_2_-DL, and SrTi_2_O_3_) are presented by (E_vib_-TS)^TiO2-SL^ – (E_vib_-TS)^SrO-SL^; (E_vib_-TS)^TiO2-DL^ – (E_vib_-TS)^SrO-SL^; and (E_vib_-TS)^SrTi2O3^ – (E_vib_-TS) ^SrO-SL^, respectively. All energies are shown for a (2×1) surface area for direct comparison.

**Note S10. Proposed process of SrTi_2_O_3_ and TiO_2_-DL surface stabilization on STO (001) surface**

It is reported that, when the TiO_2_-SL is annealed at high *P*_O2_, it becomes TiO_2_-rich surface without containing Sr ions on it, which is identified to be TiO_2_-DL^[4,28,33]^. In contrast, when annealed at low *P*_O2_, Sr ions are observed on the surface^[5,18,34–36]^. During the annealing at low *P*_O2_, the Sr coverage on the surface increases with ~1 eV peak-shift of the Sr core-level spectra which is previously interpreted as an effect of the elongated Sr-oxide formed on the TiO_2_-rich surface^[5]^. Based on our Simulation of XPS spectra of Sr 3*d* core-level, we conclude that the observed large blue shift should originate from SrTi_2_O_3_ surface. Based on the experimental observations and the analysis of the peak-shift simulation, we propose a process of how the surface is reconstructed during the annealing depending on *P*_O2_ (see Figure S11). For high *P*_O2_, all surface area becomes TiO_2_-DL without Sr atom on the surface. For low *P*_O2_, some Sr atoms remain on the surface and the surface area containing the Sr atoms becomes SrTi_2_O_3_, while the surface area not containing Sr atoms becomes TiO_2_-DL, forming a mixture of SrTi_2_O_3_ and TiO_2_-DL areas. Further annealing at low *P*_O2_ increases the SrTi_2_O_3_ area by replacing O atoms on the TiO_2_-DL area with Sr atoms (see Figure 1d-i in main article), and thus the Sr coverage on the surface. We examine the validity of our proposed process by comparing the stabilities of TiO_2_-DL and SrTi_2_O_3_ (Figure S12). With high *P*_O2_, TiO_2_-DL is more stable than SrTi_2_O_3_ for all *T* investigated. With low *P*_O2_, TiO_2_-DL is more stable than SrTi_2_O_3_ at low *T*, whereas SrTi_2_O_3_ becomes more stable than TiO_2_-DL at high *T*, consistent with the experimental reports^[5,34]^. The transition temperature between the two surfaces decreases as *P*_O2_ decreases, consistent with experiments^[5,34]^.

**
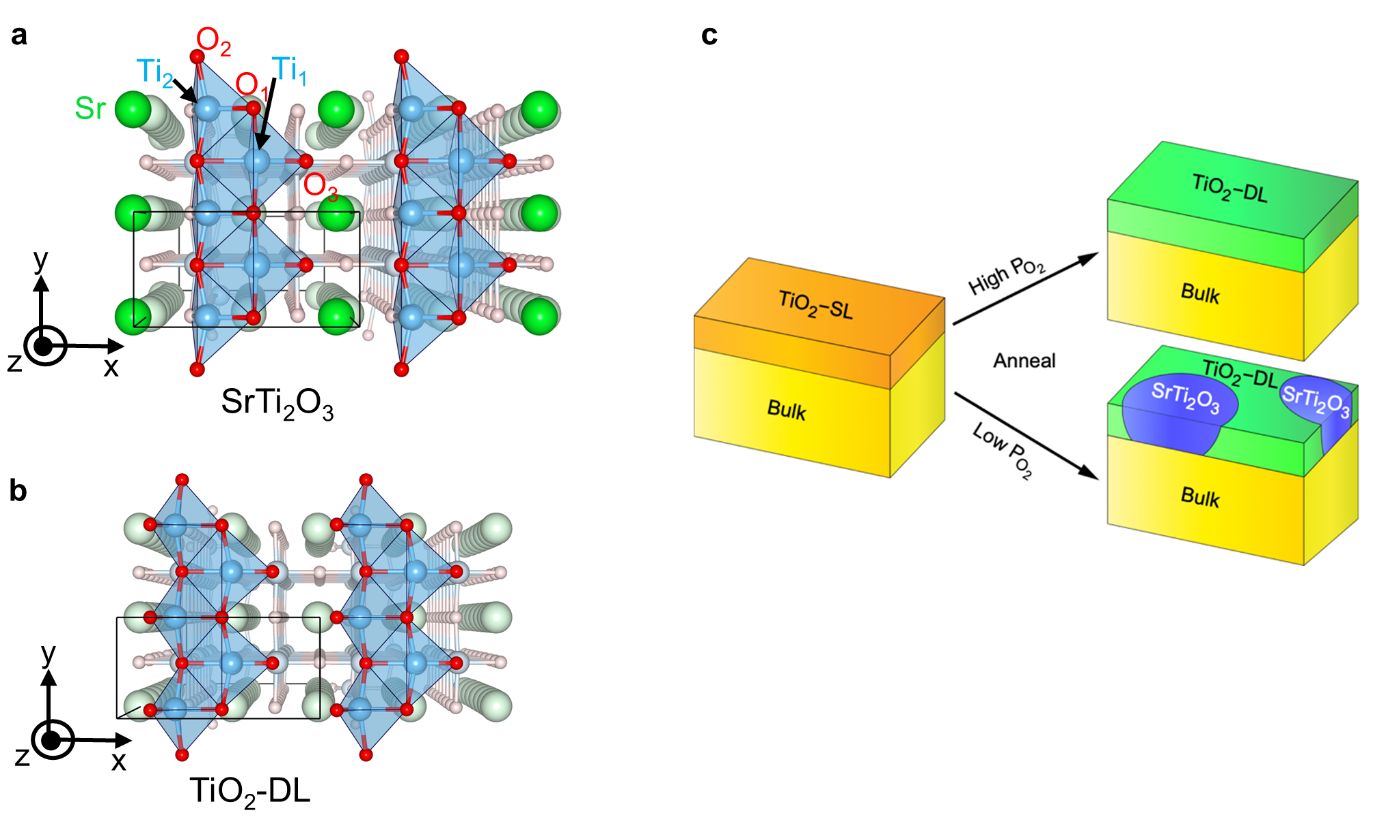
**

**Figure S14**. Proposed process of SrTi_2_O_3_ and TiO_2_-DL surface stabilization on STO (001) surface. The atomic structures of (a) SrTi_2_O_3_ and (b) TiO_2_-DL surface’s top view are shown. The *z*-direction is normal to the surface. The green, cyan, and red spheres represent the Sr, Ti, and O atoms, respectively, and the Ti-centered octahedrons are shaded in cyan. The atoms not on the surface are blurred in order to focus on the surface structure, and black rectangles represent the (2×1) surface unit cells. Schematic representation of the proposed process of SrTi_2_O_3_ and TiO_2_-DL surface stabilization. (c) Proposed process of SrTi_2_O_3_ and TiO_2_-DL surface stabilization is shown schematically. TiO_2_-DL surface could be achieved by annealing the chemically etched TiO_2_-SL at high *P*_O2_. SrTi_2_O_3_ could evolve and be mixed with TiO_2_-DL at low *P*_O2_


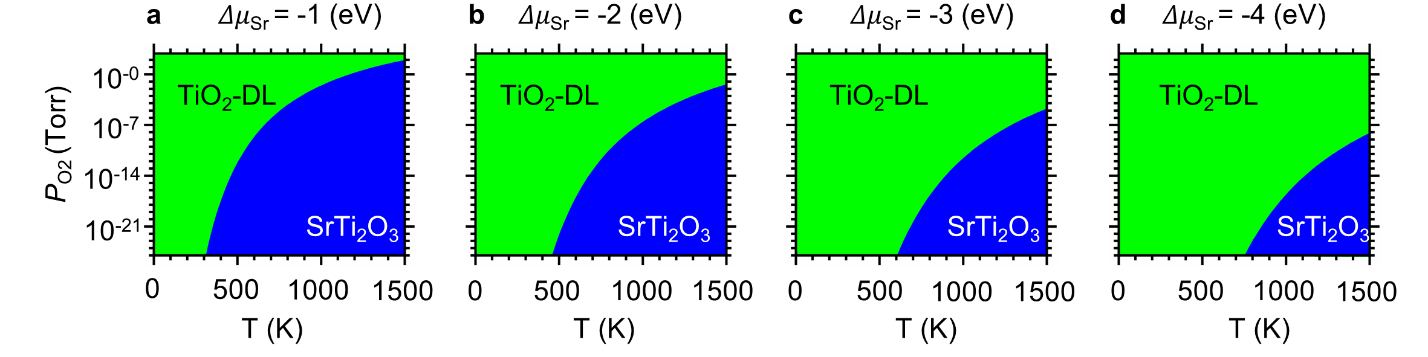


**Figure S15**. Comparison of the stabilities of SrTi_2_O_3_ and TiO_2_-DL at various *T* and 𝛥𝜇_Sr_. Diagram shows more stable surfaces between SrTi_2_O_3_ and TiO_2_-DL as a function of *T* and *P*_O2_ with 𝛥𝜇_Sr_ = –1 eV (a), –2 eV (b), –3 eV (c), and –4 eV (d). Blue represents the region where SrTi_2_O_3_ is more stable than TiO_2_-DL, while green represents the region where TiO_2_-DL is more stable than SrTi_2_O_3_.

**Note S11. Electronic structures of SrTi_2_O_3_ surface with different magnetic configurations and different U**

The anisotropic metallic behavior of the newly found SrTi_2_O_3_ surface due to the dispersive band crossing the Fermi level in one direction while flat in the other direction (see Figure 4 in main article) do not depend on the magnetic configuration (Figure S13 and S14) or calculation parameters such as Coulomb repulsion U (Figure S15). The dispersive band, connecting the bonding and antibonding states between the surface Ti, is originated from the characteristic surface structure and the subsequent electron hopping between the atoms regardless of the calculation parameters (Figure S13, S14, and S15).


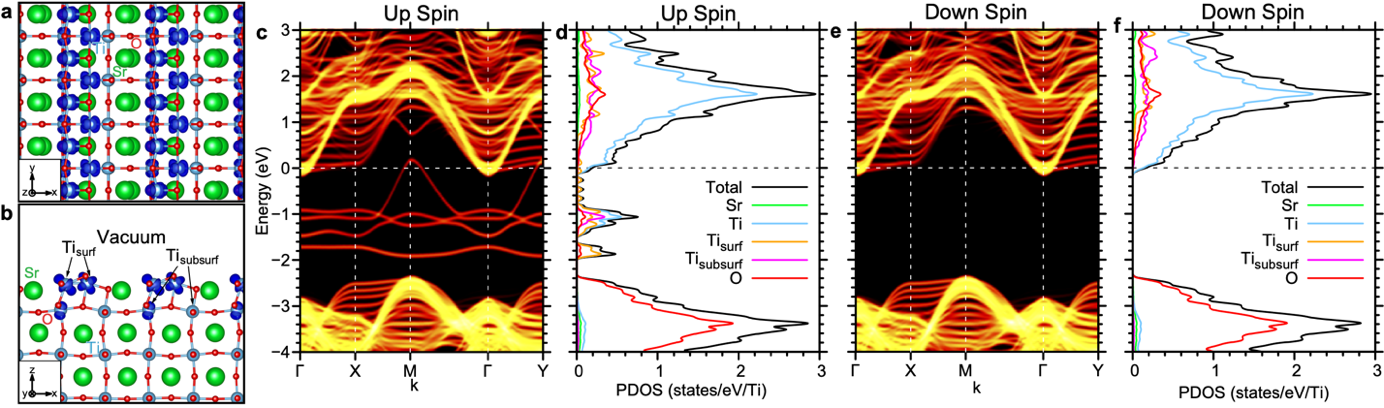


**Figure S16.** Electronic structures of SrTi_2_O_3_ surface with the ferromagnetic configuration. a-b) The electronic structures of the SrTi_2_O_3_ surface for the ferromagnetic configuration. The ferromagnetic configuration has 7.3 meV/unit area higher energy than the ferrimagnetic ground state. The spin density of the ferromagnetic configurations is shown with the atomic structure. The isosurfaces of the spin density, which is defined as the difference between the charge densities of the majority (up) and minority (down) spin components, are shaded in blue. c-f) The electronic band structures and the projected density of states of the surface. The electronic structures for the majority (c,d) and minority (e,f) spins of the ferromagnetic configuration are presented. In (c-f), the Fermi energy is set to be zero and marked with horizontal dashed lines. In (c) and (e), the bands are unfolded with respect to the first Brillouin zone of the (1×1) surface unit cell, and the denoted high-symmetry points are Γ=0, X=π/*a* $\hat{x}$, Y=π/*a* $\hat{y}$, and M=π/*a* ($\hat{x}$ + $\hat{y}$) with the optimized lattice constant *a* = 3.972 Å. In (d) and (f), the total density of states is plotted by black lines, and the density of states projected onto Sr, Ti, and O atoms are plotted by green, cyan, and red lines, respectively. The densities of states projected onto Ti atoms on the surface (denoted as Ti_surf_) and those on the sub-surface (denoted as Ti_subsurf_) are separately plotted by yellow and magenta lines, respectively.


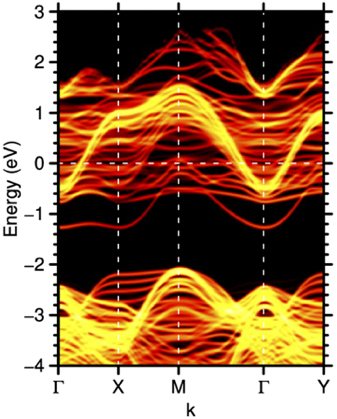


**Figure S17.** Electronic band structure of SrTi_2_O_3_ surface with nonmagnetic configuration. The electronic band structure of the SrTi_2_O_3_ surface for nonmagnetic configuration is shown. The Fermi energy is set to be zero and marked with horizontal dashed lines. The bands are unfolded with respect to the first Brillouin zone of the (1×1) surface unit cell, and the denoted high-symmetry points are Γ=0, X=π/*a* $\hat{x}$, Y=π/*a* $\hat{y}$, and M=π/*a* ($\hat{x}$ + $\hat{y}$) with the optimized lattice constant *a* = 3.972 Å.


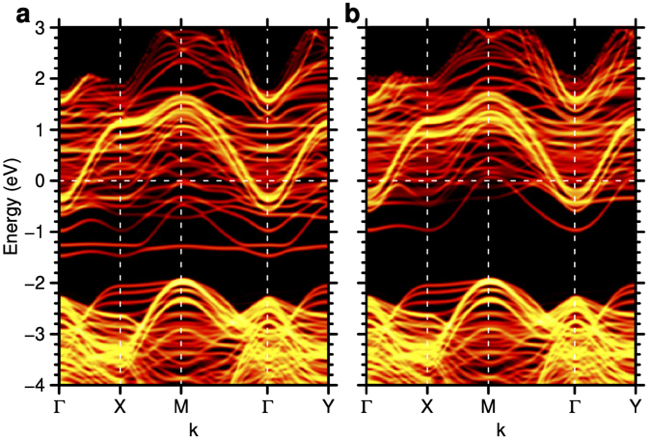


**Figure S18.** Electronic band structure of SrTi_2_O_3_ surface without considering Coulomb repulsion U. The electronic band structure of the SrTi_2_O_3_ surface with U = 0 is shown. The electronic band structures of the surface for the majority (a) and minority (b) spins are presented. The Fermi energy is set to be zero and marked with horizontal dashed lines. The bands are unfolded with respect to the first Brillouin zone of the (1×1) surface unit cell, and the denoted high-symmetry points are Γ=0, X=π/*a* $\hat{x}$, Y=π/*a* $\hat{y}$, and M=π/*a* ($\hat{x}$ + $\hat{y}$) with the optimized lattice constant *a* = 3.972 Å.

**References for Supporting Information**

[1] S. Piskunov, E. Heifets, R. I. Eglitis, G. Borstel, *Comput. Mater. Sci.* **2004**, *29*, 165.

[2] J. Padilla, D. Vanderbilt, *Surf. Sci.* **1998**, *418*, 64.

[3] E. Heifets, R. I. Eglitis, E. A. Kotomin, J. Maier, G. Borstel, *Phys. Rev. B* **2001**, *64*, 235417.

[4] N. Erdman, K. R. Poeppelmeier, M. Asta, O. Warschkow, D. E. Ellis, L. D. Marks, *Nature* **2002**, *419*, 55.

[5] T. Matsuda, Y. Yoshida, K. Mitsuhara, Y. Kido, *J. Chem. Phys.* **2013**, *138*, 244705.

[6] Y. Liang, D. A. Bonnell, *Surf. Sci.* **1993**, *285*, 510.

[7] B. Cord, R. Courths, *Surf. Sci.* **1985**, *162*, 34.

[8] P. A. W. Van Der Heide, Q. D. Jiang, Y. S. Kim, J. W. Rabalais, *Surf. Sci.* **2001**, *473*, 59.

[9] F. Silly, D. T. Newell, M. R. Castell, *Surf. Sci.* **2006**, *600*, 219.

[10] M. R. Castell, *Surf. Sci.* **2002**, *505*, 1.

[11] Q. D. Jiang, J. Zegenhagen, *Surf. Sci.* **1999**, *425*, 343.

[12] S. H. Phark, Y. J. Chang, T. Won Noh, *Appl. Phys. Lett.* **2011**, *98*, 161908.

[13] N. Erdman, L. D. Marks, *Surf. Sci.* **2003**, *526*, 107.

[14] M. Lippmaa, M. Kawasaki, A. Ohtomo, T. Sato, M. Iwatsuki, H. Koinuma, *Appl. Surf. Sci.* **1998**, *130*–*132*, 582.

[15] P. J. Møller, S. A. Komolov, E. F. Lazneva, *Surf. Sci.* **1999**, *425*, 15.

[16] T. Kubo, H. Nozoye, *Surf. Sci.* **2003**, *542*, 177.

[17] N. Erdman, O. Warschkow, M. Asta, K. R. Poeppelmeier, D. E. Ellis, L. D. Marks, *J. Am. Chem. Soc.* **2003**, *125*, 10050.

[18] T. Kubo, H. Orita, H. Nozoye, *Phys. Chem. Chem. Phys.* **2011**, *13*, 16516.

[19] D. T. Newell, A. Harrison, F. Silly, M. R. Castell, *Phys. Rev. B* **2007**, *75*, 205429.

[20] Q. Jiang, J. Zegenhagen, *Surf. Sci.* **1996**, *367*, L.

[21] C. H. Lanier, A. Van De Walle, N. Erdman, E. Landree, O. Warschkow, A. Kazimirov, K. R. Poeppelmeier, J. Zegenhagen, M. Asta, L. D. Marks, *Phys. Rev. B* **2007**, *76*, 045421.

[22] H. Tanaka, T. Matsumoto, T. Kawai, S. Kawai, *Surf. Sci.* **1994**, *318*, 29.

[23] T. Kubo, H. Nozoye, *Phys. Rev. Lett.* **2001**, *86*, 1801.

[24] I. Shiraki, K. Miki, *Surf. Sci.* **2011**, *605*, 1304.

[25] M. Naito, H. Sato, *Phys. C Supercond. its Appl.* **1994**, *229*, 1.

[26] D. M. Kienzle, A. E. Becerra-Toledo, L. D. Marks, *Phys. Rev. Lett.* **2011**, *106*, 27.

[27] C. S. Guo, K. Hermann, Y. Zhao, *J. Phys. Chem. C* **2014**, *118*, 25614.

[28] S. Cook, L. D. Marks, *J. Phys. Chem. C* **2018**, *122*, 21991.

[29] A. Togo, L. Chaput, I. Tanaka, *Phys. Rev. B* **2015**, *91*, 094306.

[30] Y. Wang, J. J. Wang, W. Y. Wang, Z. G. Mei, S. L. Shang, L. Q. Chen, Z. K. Liu, *J. Phys. Condens. Matter* **2010**, *22*, 202201.

[31] E. Heifets, E. A., Y. A., S. Piskunov, J. Maier, in *Thermodyn. - Interact. Stud. - Solids, Liq. Gases*, InTech, **2011**.

[32] E. Heifets, J. Ho, B. Merinov, *Phys. Rev. B* **2007**, *75*, 155431.

[33] R. Herger, P. R. Willmott, O. Bunk, C. M. Schlepütz, B. D. Patterson, B. Delley, *Phys. Rev. Lett.* **2007**, *98*, 076102.

[34] T. Ohnishi, K. Shibuya, M. Lippmaa, D. Kobayashi, H. Kumigashira, M. Oshima, H. Koinuma, *Appl. Phys. Lett.* **2004**, *85*, 272.

[35] D. Kobayashi, H. Kumigashira, M. Oshima, T. Ohnishi, M. Lippmaa, K. Ono, M. Kawasaki, H. Koinuma, *J. Appl. Phys.* **2004**, *96*, 7183.

[36] K. Szot, W. Speier, U. Breuer, R. Meyer, J. Szade, R. Waser, *Surf. Sci.* **2000**, *460*, 112.
